# Predictors of social intermediate factors associated with sexual quality of life of women: systematic review and meta-analysis

**DOI:** 10.1186/s12905-024-02899-2

**Published:** 2024-01-24

**Authors:** Marzieh Bagherinia, Mahrokh Dolatian, Zoherh Mahmoodi, Giti Ozgoli, Hamid Alavi Majd

**Affiliations:** 1 Midwifery and Reproductive Health Research Center, Department of Midwifery and Reproductive Health, School of Nursing and Midwifery, Shahid Beheshti University of Medical Sciences, Tehran, Iran; 2 Social Determinants of Health Research Center, Alborz University of Medical Sciences, Karaj, Iran; 3 Department of Biostatistics, School of Allied Medical Sciences, Shahid Beheshti University of Medical Sciences, Tehran, Iran; 4 Tehran, Iran

**Keywords:** Sexual health, Social determinants of health, Women, Systematic review and meta-analysis

## Abstract

**Background:**

Sexual problems and diminished sexual quality of life can adversely affect the physical, psychological, and emotional health of women. The present study was done to determine the social intermediate factors of health associated with sexual quality of life in women of reproductive age.

**Design:**

Systematic review and Meta-analysis.

**Data sources:**

Embase, Web of Science, PubMed/Medline (NLM), ProQuest, and CENTRAL.

**Eligibility criteria:**

Observational studies (cross-sectional, case-control, cohort) from 2010 to 2022 with no language constraints were included. The sexual quality of life, as the main variable of the study, has been evaluated using Symonds women’s sexual quality of life scale (SQOL-F). The health social determinants intermediate factors based on WHO model were considered as exposure variables.

**Data extraction and synthesis:**

The data of included studies were extracted by two independent persons through a researcher-made checklist according to the study aims. Quality assessment of studies was done using Newcastle-Ottawa instrument. R software (Version R-4.2.1) were used for meta-analysis. Publication bias was evaluated by Egger & Begg tests. Sensitivity analysis was done through one-out remove approach.

**Results:**

Eventually, 15 studies were eligible to be included in this systematic review and meta-analysis. Variables of depression, quality of marital relation, body image, self-esteem, physical activity, and sexual function were among the health social intermediate factors associated with sexual quality of life. Publication bias had no effect on the obtained results; no study affecting the results was found through sensitivity analysis.

**Conclusion:**

Considering the relationship between modifiable factors and sexual quality of life, it seems that identification of these factors can be an important step towards designing interventional studies to help women experience enhanced sexual quality of life.

**Supplementary Information:**

The online version contains supplementary material available at 10.1186/s12905-024-02899-2.

## Background

Sexual quality of life is the person’s assessment of positive and negative aspects of their sexual life and their response to this assessment [1]. Sexual quality of life is tightly interwoven with the extent of satisfaction with life and general level of quality of life [2]. Low Sexual quality of life can reflect the health status and general quality of life [3]. Sexual problems and diminished sexual quality of life can adversely affect the physical, psychological, and emotional health of women [4]. In addition to dissociation of martial life, it can also be involved in emergence of sexual rape, as well as psychological diseases and crimes [5]. Indeed, high quality and satisfactory sexual life is a key component for women’s well fare [6]. Thus, identifying factors affecting sexual quality of life can be important. Meanwhile, sexual issues, as a component of health, can be a multi-dimensional phenomenon, not only affected by biomedical factors but also by psychological, behavioral, and social factors [7].

People’s health and its different dimensions are issues whose role in enhancing human development indices is undeniable. As such, identifying factors causing inequalities in health is regarded a priority of healthcare [8]. The human right’s international charter has especially emphasized movement towards equality in health through capturing health social determinants [9]. Various models have been presented for indicating the mechanisms of social determinants affecting health [10]. The social determinants commission of world health organization (WHO) has presented a model to cover all previous models. Based on this model, intermediate factors are a group of health social determinants, which specify differences in exposure and vulnerability with high-risk conditions for health. These include life conditions, access to food and healthcare services, psychosocial factors (psychological status), behavioral factors, lifestyle and social support, barriers against choosing a healthy life, and violence [11]. Studies have reported various factors including age [12], duration of marriage, marital relationships [13], any chronic disease [14], personality traits, depression [15], economic status [16], and the value-cultural context of the society [17] as factors associated with sexual quality of life. However, based on our search, no study was found to have exhaustively examined factors associated with sexual quality of life in women within the specific framework of health social determinants.

Since sexual quality of life plays a key role in the family and society’s health [18], and elimination of inequalities in health areas necessitates understanding variables, mechanisms, and their interrelationships. The present study was done with the aim of collating and concluding the current knowledge on the intermediate factors of health social determinants associated with sexual quality of life of women. The question of this review study is, “Which intermediate factor of health social determinants based on the WHO model is associated with sexual quality of life of women?”

## Methods

### Aim

This systematic and meta-analysis was conducted to determine the social intermediate factors of health associated with sexual quality of life in women of reproductive age.

The model used to write this systematic review study was world health organization’s model of social determinants of health [11].

### Search methods

Search was done in six databases of PubMed/Medline (NLM), Embase, Web of Science, Scopus, ProQuest, and CENTRAL from 2010 to 2022 with no language constraints systematically. For non-English papers, translation to English was done. The keywords of the two main components of “sexual quality of life” and “health social intermediate factors” were found through Mesh system in PubMed and Emtree in Embase database along with suitable synonyms. Through AND and OR operators, these words were merged with each other, and the search syntax was first prepared for PubMed. Next, this syntax was adapted for other databases. In addition, manual search of the included studies was done to find similar studies (Additional file 1).

### Inclusion/exclusion criteria

The primary aim of this study was to determine the social intermediate factors associated with women’s sexual quality of life. Accordingly, observational studies (cohort, case-control, and cross-sectional) were included. Qualitative studies, clinical trials, theses, posters, letters to editor, systematic review and meta-analyses studies were excluded. The studied population consisted of women of reproductive age (18-45 years), non-pregnant or at least 1 year past their pregnancy, no chronic disease (hypertension, diabetes, cardiovascular disease, asthma, kidney diseases, …), and different types of cancer. The sexual quality of life, as the main variable of the study, has been evaluated using Symonds women’s sexual quality of life scale (SQOL-F). The health social determinants intermediate factors based on WHO model [11] were considered as exposure variables including lifestyle, depression, anxiety, stress, social support, physical activity, self-esteem, body image, violence, high-risk behaviors, access to healthcare services, sexual function, and quality of marital relation. Assessment of intermediate factors was done using the following questionnaires: Miller-Smith lifestyle questionnaire (LSQ), beck depression inventory (BDI), hospital anxiety and depression scale (HADS) for anxiety and depression variables, Cohen’s perceived stress (PSS-14), perceived social support questionnaire Zimet (MPSS), Rosenberg self-esteem scale, body image scale (BIS) or female genital self-image scale (FGSIS-I), domestic violence or sexual violence, female sexual function index (FSFI), marital intimacy needs questionnaire (MINQ) or dyadic adjustment scale (DAS), high-risk behaviors (cigarette smoking, alcohol and tobacco consumption), physical activity and access to healthcare services through demographic questionnaire was captured through yes/no question. The secondary aim in this study was to determine any possible relationship between duration of marriage and sexual quality of life.

### Data extraction

The data of included studies were extracted by two independent persons through a researcher-made checklist according to the study aims. This information included author’s name, year of publication, place of study, mean score of sexual quality of life, effect size, sample size, mean age of subjects, type of study (cross-sectional, cohort, case-control), sampling method (random or nonrandomized), measured variables, utilized instruments, and quality assessment score of the primary studies.

R software (Version R-4.2.1) along with Metacore function belonging to meta package (Version 5.5-0) were used for meta-analysis. Correlation coefficients were converted to Z fisher scores and then merged. These values were calculated through the Metacore function. Considering the expected diversity in primary studies, Random model and inverse variance type were used. To indicate the results, forest plot was used with confidence interval 95%. I^2^ index and chi^2^ test were used for assessing the heterogeneity of studies. Accordingly, I^2^ lower than 25% functioned as low heterogeneity, 25-50% as moderate, 50-75% as high, and more than 75% as very severe heterogeneity (considerable heterogeneity) [19]. Subgroup analysis method was used for finding possible sources of heterogeneity in cases of I^2^ above 75%. Correlation coefficient with 95% confidence interval was used for determining the relationship of social intermediate factors and sexual quality of life with interpretive level, defined as 0.1-0.29 weak level, 0.30-0.49 moderate level, and 0.50 and above as strong level [20]. Begg & Egger tests were used for assessing publication bias of studies through metabias function belonging to meta package. In all analyses, significance level was considered *p* <  0.05. However, for Begg & Egger tests, due to the limited number of studies, significance level was considered *p* <  0.10. Sensitivity analysis was done to assess the effect of each study on the overall outcome using one-out remove approach, and it was calculated through metanif function from meta package.

### Methodological quality assessment of studies

Two persons (M.B, Z.M) independently evaluated the primary studies in terms of methodological quality using Newcastle-Ottawa instrument. It is a specific tool for measuring the quality of observational studies. Studies based on this instrument are examined in terms of quality of study design, data collection method, sample registration process, response rate, generalizability of results, and statistical analyses. For cohort, case-control, and cross-sectional studies, this instrument has a specific version. The scoring of the items of this instrument can be as no star (score 0), one star (score 1), and two stars (score 2). The maximum score would be 10 stars, where studies with six stars (score) and above were considered as those with a high methodological quality (Additional file 2) [21]. In assessing the quality of studies, screening and extraction of data were done by two independent persons. In cases of disagreements, two researchers discussed, resolved disagreements, and created a one-sheet report of findings at a meeting.

## Results

### Search outcomes

**Fig. 1:**
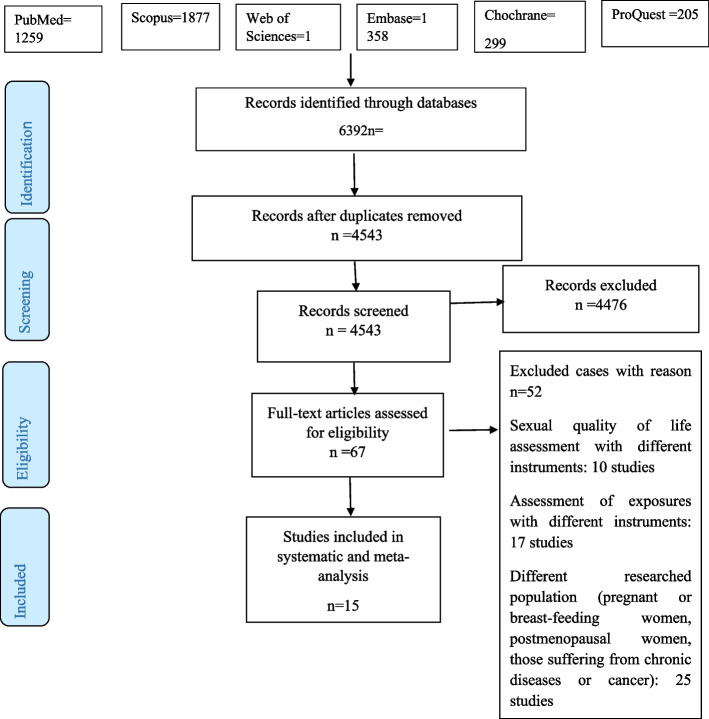
Flowchart of study

In response to the search across databases of PubMed = 1259, Scopus = 1877, Embase = 1358, Web of Science = 1394, Cochrane = 299, and ProQuest = 205, overall 6392 papers were found and inputted into EndNote software. After removing duplicates, 4543 studies were investigated by two independent persons in two separate stages. The first stage involved examining the title and abstract of studies, based on which 4476 studies that did not meet the inclusion criteria and were excluded. Regarding the remaining 67 studies, their full text was re-examined in terms of inclusion and exclusion criteria. After the second stage, eventually 15 studies met the inclusion criteria to be included in this systematic review and meta-analysis, based on which data extraction was done (Fig. 1).

### Study characteristics

The included studies had sample sizes of at least 88 and at most 800 subjects. The total sum of all study samples was 3878. The overall mean age of the women based on included studies was 33.6 ± 3.4 years, and the overall mean score of the sexual quality of life was 71.11 ± 8.7. Regarding design of conducting primary studies, two had been done as case-control [22, 23], one as cohort [24], and 12 as cross-sectional [25–36]. From among the cross-sectional ones, only six of them had random sampling method [25, 29–32, 34]. In the included studies, 7 have been conducted in Iran, 6 in Turkey, 1in Spain and 1 study in France. The full text of a study was in Persian [27], one in Turkish [29], and other in English. Methodological quality assessment of studies based on Newcastle-Ottawa scale was high in five studies (score above 6) [25, 26, 31–33] (Table 1 and Additional file 2).

**Table 1:** Characteristics of included studies

Author, Year/ Country of study	Design study	Sample size	Mean of age	Mean of SQOL^a^	Controlled variables and types of tools used	Type of analysis	Main result (Variables related to the quality of sexual life)	Quality assessment score (NOS^b^)
Sheikhan et al., 2019/ Iran [25]	Cross-sectional (random)	800	–	–	Demographic: age, education, age at marriage, age at monarch, duration of marriage, age at first pregnancy, having private bedroom, smoking and addicted at spouse, drinking alcohol Stress (stress perceived Cohen), Sexual violence (self-designed), Sexual quality of life (SQOL-F)	Bivariate analysis	Sexual violence: *r* = −0.502, *P* = - Stress: *r* = − 0.228, *P* = - Alcohol used:- Duration of marriage:-	8
Eftekhar et al., 2019/ Iran [22]	Case-control	150 (50 case, 100 control)	37.8 ± 9.4	79.5 ± 20.6	Demographic: age, education, duration of marriage, body mass index (BMI) Sexual function (Female Sexual Function Index (FSFI)), Sexual quality of life (SQOL-F), Female genital self-image (Female genital self-image scale (FGSIS-I))	Multivariate analysis	Sexual function: *r* = 0.543 Female Genital Self-Image: *r* = 0.121 Duration of marriage: *r* = 0.187	5
Türkben Polat and Kaplan Serin, 2021/ Turkey [26]	Cross-sectional (nonrandom)	90	32.9 ± 7.7	50.4 ± 10.2	Demographic: age, education, duration of marriage, parity, Body mass index (BMI), occupation, income, family planning, smoking, alcohol used, weight satisfaction, physical activity, efforts to lose weight, meditation Self-esteem (Rosenberg self-esteem scale), Sexual quality of life (SQOL-F)	Bivariate analysis	Self-esteem: *r* = 0.286, *P* < 0.01 Smoking:- Alcohol used:- Physical activity: *r* = 0.236 Duration of marriage: *r* = − 0.061	6
Samimi et al., 2016/ Iran [27]	Cross-sectional (nonrandom)	121	32.4 + 7.5	80.1 ± 19.7	Demographic: age, number of members Family, education, education of spouse, age of spouse, duration of marriage Variables related to health: quantity and quality of sleep, Body mass index (BMI), physical activity Factors related to work: Work, type of work system, having a second job, overtime, hours work per week, type of work activity, occupational accidents Sexual quality of life (SQOL-F)	Bivariate analysis	Physical activity: *r* = 0.195 Duration of marriage: *r* = −0.381	4
Tugut et al., 2021/ Turkey [28]	Cross-sectional (nonrandom)	100	38.7 ± 8.9	76.8 ± 15.3	Demographic: age, education, occupation, income, family type, number of children, place of residence, social support from spouse and support from other family members, economic status, average length of marriage Depression (Beck Depression Inventory (BDI)), General health (GH-28), Sexual quality of life (SQOL-F)	Bivariate analysis	Depression: *r* = −0.52, *P* = 0.00 General health: *r* = − 0.47, *P* = 0.00 Duration of marriage: -	4
Tav et al., 2018/ Turkey [29]	Cross-sectional (random)	162	38.8 ± 5.4	–	Demographic: age, place of residence, education, income, age at marriage, Work Dyadic adjustment (dyadic adjustment scale (DAS)), Violence, Sexual quality of life (SQOL-F)	Bivariate analysis	Dyadic adjustment: *r* = 0.576, *p* < 0.001 Violence:-	5
Taskin Yilmaz et al., 2019/ Turkey [30]	Cross-sectional (random)	538	35.5 ± 8.7	83.3 ± 16.4	Demographic: body weight preference, body weight perception, healthy nutritional status, exercise, general health perception Body Image (BIS), Sexual quality of life (SQOL-F)	Bivariate analysis	Body image: *r* = 0.381, *P* = 0.00 Exercise: -	4
Shahraki et al., 2018/ Iran [31]	Cross-sectional (random)	264	32.9 ± 7.2	84.8 ± 18.9	Demographic: age, partner age, duration of marriage, pervious abortion Depression (Beck Depression Inventory (BDI)), Sexual function (Female Sexual Function Index (FSFI)), Sexual quality of life (SQOL-F)	Multivariate analysis	Sexual function: *r* = 0.59, *P* < 0.001 Depression: *r* = − 0.49, *P* < 0.001 Duration of marriage: *r* = − 0.03	6
Haghi et al., 2018/ Iran [32]	Cross-sectional (random)	475	–	–	Demographic: age, weight, height, age of husband, duration of marriage, living with husband’s family, the history of boyfriend, relationship before marriage, job, working in night shifts, education, income Perceived sexual characteristics (self-designed), Sexual behavior variables (self-designed), Sexual function (Female Sexual Function Index (FSFI)), Marital intimacy (Marital Intimacy Needs Questionnaire (MINQ)), Sexual quality of life (SQOL-F)	Bivariate analysis	Sexual function: *r* = 0.39, P < 0.001 Marital intimacy: *r* = 0.350, *P* < 0.001	7
Velayati et al., 2021/ Iran [33]	Cross-sectional (nonrandom)	236	27.9 ± 5.7	56.8 ± 20.2	Demographic: age, education, occupation, income, house status, age of husband, Husband’s education, information source, having child Self-esteem (Rosenberg self-esteem scale), Depression and anxiety (Hospital Anxiety and Depression Scale (HADS)), Sexual quality of life (SQOL-F)	Bivariate analysis	Self-esteem: *r* = 0.54, *P* < 0.001 Anxiety: *r* = − 0.48,P < 0.001 Depression: *r* = − 0.47,*P* < 0.001	7
Panahi et al.,2021/ Iran [34]	Cross-sectional (random)	420	33.1 ± 4.6	59.7 ± 19.2	Demographic: age, education, employment status, age of first child, age of spouse, spouse’s educational, duration of marriage, age at marriage, number of weekly sexual intercourses, use of contraceptives Sexual function (Female Sexual Function Index (FSFI)), Sexual quality of life (SQOL-F)	Multivariate analysis	Sexual function: *r* = 0.306 Duration of marriage: *r* = 0.101	5
Alcalde et al., 2021/ Spain [35]	Cross-sectional (nonrandom)	173	37.8 ± 5.4	80.2 ± 4.3	Demographic: age, education, Body mass index (BMI), age of spouse, spouse’s educational, heavy menstrual bleeding, dysmenorrhea, dyspareunia Dyadic adjustment (dyadic adjustment scale (DAS)), Sexual quality of life (SQOL-F)	Bivariate analysis	Dyadic adjustment: *r* = 0.008	5
Yuksekol et al., 2021/ Turkey [36]	Cross-sectional (nonrandom)	135	31.2 ± 5.9	65.6 ± 26.8	Demographic: Age, education, age at monarch, duration of marriage, income, work, living place, family type, duration of infertility, vaccination Female genital self-image (Female Genital Self-Image Scale (FGSIS)), Dyadic adjustment (dyadic adjustment scale (DAS)), Sexual quality of life (SQOL-F)	Bivariate analysis	Female genital self-image: *r* = 0.618, P = 0.00 Dyadic adjustment: *r* = 0.542, *P* = 0.00 Duration of marriage: *r* = 0.934. *P* = 0.007	5
Brunault et al., 2015/ France [24]	Cohort	126	–	–	Demographic: age, marital status, previous maximal body mass index (BMI), current BMI, history of previous bariatric surgery Binge eating severity (using the Bulimic Investigatory Test, Edinburgh = BITE) Depression (Beck Depression Inventory (BDI)), Sexual quality of life (SQOL-F)	Bivariate analysis	Depression: *r* = − 0.60, *P* < 0.01 Binge eating severity: *r* = − 0.47, P < 0.01	4
Telli et al., 2020/ Turkey [23]	Case-control	176 (88 case, 88 control)	35.7 ± 6.2	80.1 ± 21.4	Demographic: Age, education, income, Employment status, Child presence Dyadic adjustment (dyadic adjustment scale (DAS)), Sexual quality of life (SQOL-F)	Bivariate analysis	**Result on Control group (health)** Dyadic adjustment: *r* = 0.500, P < 0.01	4

^a^ Sexual quality of life

^b^ Newcastle-Ottawa Scale

*r* = Correlation coefficient with 95% confidence interval

### Meta-analysis results

#### Depression and quality of sexual life

**Fig. 2:**
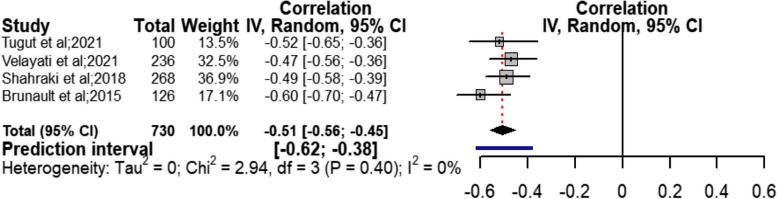
Forest Plot correlation coefficient with 95% confidence interval between depression and quality of sexual life

The results of meta-analysis on variable of depression as one of the health social intermediate determinants with four studies [24, 28, 31, 33] showed an almost strong and negative relationship with sexual quality of life (*r* = − 0.51; CI95% = − 0.56 to − 0.45; chi^2^ = 2.94; I^2^ = 0%; *P* = 0.40). The confidence interval for this correlation ranged from moderate to strong with low heterogeneity (Fig. 2). Begg test with *P* = 0.734, Z = -0.34, and Egger with t = − 1.39, *P* = 0.298 indicated that the publication bias had no effect on the outcome obtained in this section. Sensitivity analysis on the relationship between variable of depression and sexual quality of life showed that elimination of any single study did not have a considerable effect on the outcome obtained from combining all studies (Table 2).

**Table 2:** Summary of sensitivity analysis on the variables related to the sexual quality of life

Omitted study	Correlation Coefficient	Confidence interval	Heterogeneity index	
			chi^2^ test (*P*-value)	I^2^
Quality of marital relation and quality of sexual life				
Yuksekol et al.;2021 [36]	0.37	0.11 to 0.58	0.006	92.1%
Haghi et al.;2018 [32]	0.42	0.15 to 0.63	0.003	93.1%
Tav et al.;2018 [29]	0.36	0.11 to 0.56	0.005	90.7%
Telli et al.;2020 [23]	0.39	0.11 to 0.60	0.005	93.0%
Alcalde et al.;2021 [35]	0.49	0.37 to 0.59	< 0.001	78.1%
All studies (without removing any study)	0.41	0.20 to 0.58	0.000	91.0%
Body image and quality of sexual life				
Eftekhar et al.;2019 [22]	0.50	0.23 to 0.69	0.0006	90.8%
Taskin Yilmaz et al.;2019 [30]	0.40	−0.16 to 0.76	0.160	96.1%
Yuksekol et al.;2021 [36]	0.26	−0.004 to 0.49	0.053	89.4%
All studies (without removing any study)	0.39	0.08 to 0.63	0.015	92.2%
Sexual function and quality of sexual life				
Eftekhar et al.;2019 [22]	0.43	0.25 to 0.56	< 0.0001	90.9%
Shahraki et al.;2018 [31]	0.40	0.36 to 0.52	< 0.0001	79.4%
Haghi et al.;2018 [32]	0.48	0.43 to 0.60	< 0.0001	91.7%
Panahi et al.;2021 [34]	0.50	0.37 to 0.62	< 0.0001	85.5%
All studies (without removing any study)	0.45	0.32 to 0.58	< 0.0001	85.5%
Depression and quality of sexual life				
Tugut et al.;2021 [28]	−0.50	−0.569 to − 0.44	< 0.0001	31.3%
Velayati et al.;2021 [33]	−0.52	− 0.59 to − 0.45	< 0.0001	3.8%
Shahraki et al.;2018 [31]	− 0.52	− 0.60 to − 0.43	< 0.0001	25.9%
Brunault et al.;2015 [24]	− 0.48	− 0.54 to − 0.42	< 0.0001	0.0%
All studies (without removing any study)	− 0.51	− 0.56 to − 0.46	0.40	0.0%
Secondary aim (Duration of marriage and sexual quality of life)				
Eftekhar et al.;2019 [22]	0.25	− 0.43 to 0.57	< 0.01	99%
Yuksekol et al.;2021 [36]	0.31	−0.22 to 0.69	< 0.01	86%
Shahraki et al.;2018 [31]	0.29	−0.38 to 0.77	< 0.01	99%
Turkben Polat and Kaplan et al.;2021 [26]	0.30	−0.37 to 0.77	< 0.01	99%
Samimian et al.; 2016	0.36	−0.26 to 0.77	< 0.01	99%
Panahi et al.;2021 [34]	0.27	−0.41 to 0.76	< 0.01	99%
All studies (without removing any study)	0.24	−0.33 to 0.68	< 0.01	99%

#### Sexual function and quality of sexual life

**Fig. 3:**
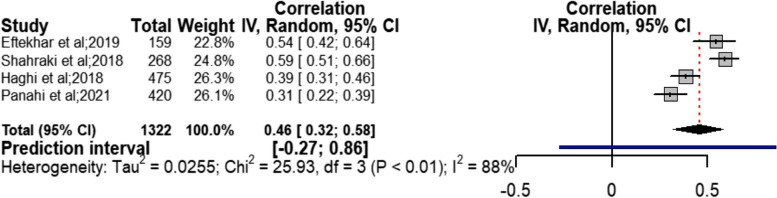
Forest Plot correlation coefficient with 95% confidence interval between sexual function and quality of sexual life

Meta-analysis of the sexual function variable out of health social intermediate factors with sexual quality of life with four studies [22, 31, 32, 34] showed a moderate and positive relationship with confidence interval ranging from moderate to strong (*r* = 0.46; CI95% = 0.32 to 0.58; chi^2^ = 25.93; I^2^ = 88%; *P* < 0.01) (Fig. 3). Considering very severe heterogeneity (I^2^ = 88%) in this section, to identify the possible factors affecting this heterogeneity, subgroup analysis was done. From among the controlled factors, the sample size variable had a greater impact compared to others on reducing the heterogeneity index (average 65.5%) with *p* < 0.01 (Table 3 and Additional file 3 (fig. S1-S4)). Accordingly, in studies with smaller sample size, a stronger correlation and reduction of heterogeneity were observed compared to overall combination of studies. The results of Begg (Z = 0.34, *P* = 0.734) and Egger (t = 1.60, *P* = 0.249) tests did not show considerable publication bias against the obtained outcome. The results of sensitivity analysis in this section showed that removal of each single study did not have a considerable effect on the outcome obtained from combining all studies (Table 2).

**Table 3:** Subgroup analysis for sexual function relationship and quality of sexual life

Subgroup variables		Number	Correlation coefficient	Confidence Interval	chi^2^ test (hetrogeneity *p*-value)	I^2^	Test for interaction (*P*-value)
Sample size	> = 400	2	0.35	0.51 to 0.63	0.18	45%	< 0.01
	< 400	2	0.57	0.27 to 0.42	0.49	0.0%	
Score quality assessment	> = 6 (High quality)	2	0.49	0.27 to 0.67	< 0.01	92%	0.67
	< 6 (low quality)	2	0.43	0.17 to 0.63	< 0.01	90%	
Study design	Cross-sectional study	3	0.43	0.25 to 0.59	< 0.01	91%	0.98
	Case control study	1	0.54	0.42 to 0.68	–	–	
Type of analysis	Bivariate analysis	1	0.39	0.31 to 0.46	–	–	
	Multivariate analysis	3	0.49	0.30 to 0.64	< 0.01	92%	0.32
All study		4	0.46	0.32 to 0.58	< 0.01	88%	–

#### Body image and quality of sexual life

**Fig. 4:**
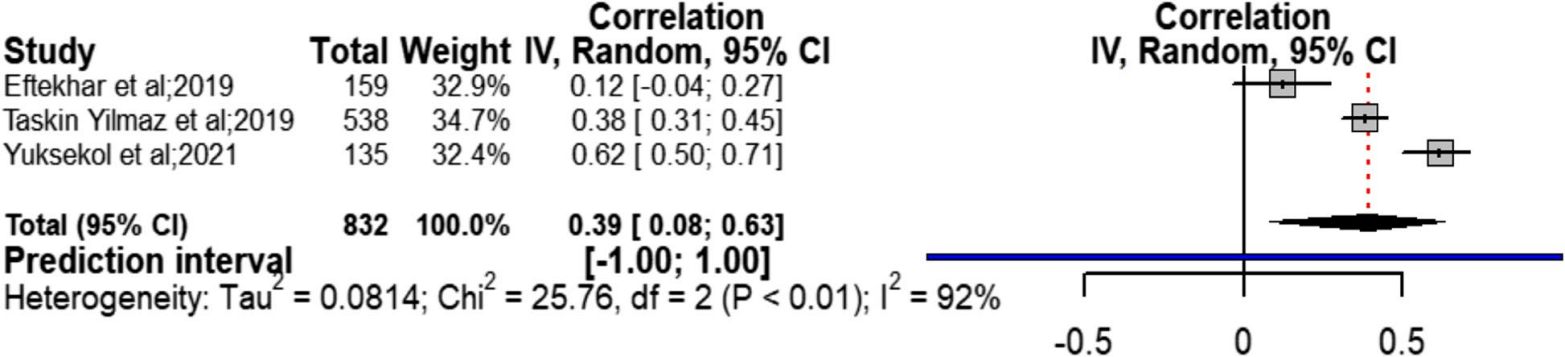
Forest Plot correlation coefficient with 95% confidence interval between body image and quality of sexual life

Meta-analysis on three studies [22, 30, 36] between the body image variable from among the health social intermediate factors and sexual quality of life showed a moderate positive relationship and confidence interval from very weak to strong and very severe heterogeneity (*r* = 0.39; CI95% = 0.08 to 0.63; chi^2^ = 25; I^2^ = 92%; *P* < 0.01) (Fig. 4). Due to limited number of studies, it was not possible to identify possible source of heterogeneity in this section. The results of Begg (Z = 0.0, *P* = 1.0) and Egger (t = 0.10, *P* = 0.939) tests did not show considerable publication bias on the obtained result. The results of sensitivity analysis also showed that removal of each single study did not have considerable effect on the outcome obtained from combining all studies (Table 2).

#### Self-esteem and quality of sexual life

**Fig. 5:**
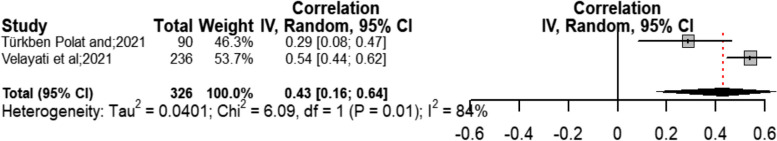
Forest Plot correlation coefficient with 95% confidence interval between self-esteem and quality of sexual life

Meta-analysis for the relationship between self-esteem variable from among health social intermediate factors with two studies [26, 33] revealed a positive moderate relationship with confidence interval of weak to strong and very severe heterogeneity with sexual quality of life (*r* = 0.43; CI95% = 0.16 to 0.64; chi^2^ = 6.09; I^2^ = 84%; *P* = 0.01) (Fig. 5). Due to limited number of studies, it was not possible to evaluate the publication bias or do sensitivity analysis.

#### Physical activity and quality of sexual life

**Fig. 6:**
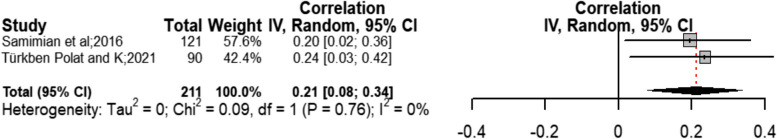
Forest Plot correlation coefficient with 95% confidence interval between physical activity and quality of sexual life

Meta-analysis on the variable of physical activity from among the health social intermediate factors with two studies [26, 27] revealed a very weak positive relationship with confidence interval ranging from very weak to moderate with sexual quality of life (r = 0.21; CI95% = 0.08 to 0.34; chi^2^ = 0.09; I^2^ = 0%; *P* = 0.76) (Fig. 6). Due to limited number of studies, it was not possible to evaluate the publication bias or do sensitivity analysis.

#### Quality of marital relation and quality of sexual life

**Fig. 7:**
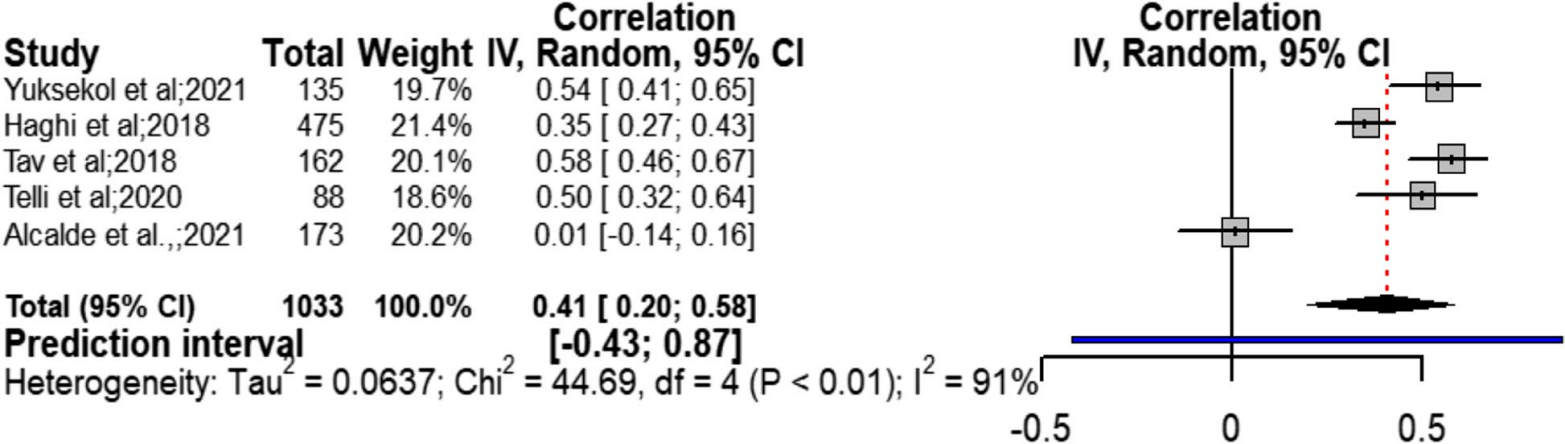
Forest Plot correlation coefficient with 95% confidence interval between quality of marital relation and quality of sexual life

Meta-analysis of the variable of quality of marital relation from among the health social intermediate variables with five studies [23, 29, 32, 35, 36] showed a moderate positive relationship with confidence interval ranging from weak to strong with sexual quality of life (r = 0.41; CI95% = 0.20 to 0.58; chi^2^ = 25; I^2^ = 91%; *P* < 0.01) (Fig. 7). Considering the value of I^2^ = 91% (very severe heterogeneity), subgroup analysis was done. The results revealed that none of the variables of sample size (*P* = 0.62), type of instrument (*000000000*0.58), quality assessment score (*P* = 0.76), and type of study design (*P* = 0.44) justified the creation of heterogeneity in the relationship between inter-couple relationships and sexual quality of life (Table 4 and Additional file 3 (fig. S5-S8)). The results of Begg (Z = 0.24, *P* = 0.806) and Egger (t = 0.51, *P* = 0.642) did not show considerable publication bias on the results obtained in this section. The results of sensitivity analysis showed that removal of every single study did not have a considerable impact on the outcome obtained from combining all studies (Table 2).

**Table 4:** Subgroup analysis for quality of marital relation and quality of sexual life

Subgroup variables		Number	Correlation coefficient	Confidence Interval	chi^2^ test (hetrogeneity *p*-value)	I^2^	(*p*-value) test for interaction
Type of scale	Dyadic Adjustment Scale	4	0.42	0.15 to 0.64	< 0.01	93%	0.58
	Marital Intimacy Questionnaire	1	0.35	0.27 to 0.43	–	–	
Score quality assessment	> = 6	1	0.35	0.27 to 0.43	–	–	0.58
	< 6	4	0.42	0.15 to 0.64	< 0.01	93%	
Study design	Crossectional study	4	0.39	0.12 to 0.60	< 0.01	93%	0.44
	Case control study	1	0.50	0.32 to 0.64	–	–	
Sample size	> = 150	2	0.47	0.22 to 0.66	< 0.01	90%	0.62
	< 150	3	0.37	0.01 to 0.64	< 0.01	94%	
All study		5	0.41	0.20 to 0.58	< 0.01	91%	–

Considering other intermediate variables of health social determinants, high-risk behavior (consumption of cigarette, alcohol, drugs of abuse) in two studies [25, 26], anxiety one study [33], stress one study [25], and variable of violence in two studies [25, 29] had a relationship with sexual quality of life. In the variables of high-risk behaviors and violence, in spite of two studies, it was not possible to calculate correlation coefficient for meta-analysis. Again, no study was found on the relationship between social support, access to healthcare services, and lifestyle variables from among the intermediate factors and sexual quality of life based on the search or inclusion/exclusion criteria (Table 1).

#### Secondary aim

**Fig. 8:**
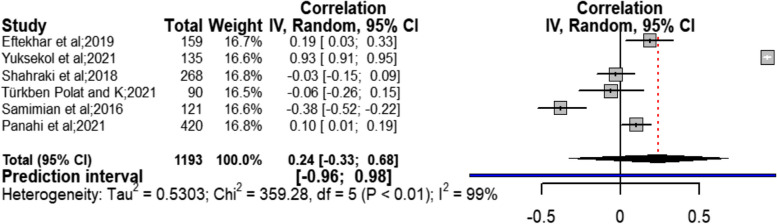
Forest Plot correlation coefficient with 95% confidence interval between duration of marriage and quality of sexual life

Meta-analysis of the relationship between duration of marriage and sexual quality of life as the secondary study aim, based on six studies [22, 26, 27, 31, 34, 36], showed that there was no significant relationship between duration of marriage and sexual quality of life (*r* = 0.24; CI95% = − 0.33 to 0.68; chi^2^ = 359.28; I^2^ = 99%; *P* < 0.01) (Fig. 8). Considering the very severe heterogeneity (I^2^ = 99%) in this section, to identify the possible factors contributing to heterogeneity, subgroup analysis was done. The results indicated that none of the variables of sample size (*P* = 0.50), type of analysis (*P* = 0.61), quality assessment score (*P* = 0.34), and type of study design (*P* = 0.85) justified creation of heterogeneity in the association between duration of marriage and sexual quality of life (Table 5 and Additional file 3 (fig. S9-S12)). The results of Begg (Z = 0.0, *P* = 1.0) and Egger (t = 0.38, *P* = 0.726) tests did not show considerable publication bias on the result obtained in this section. The result obtained from sensitivity analysis showed that removal of every single study did not considerable effect on the outcome resulting from combining all studies (Table 2).

**Table 5:** Subgroup analysis for duration of marriage and quality of sexual life

Subgroup variables		Number	Correlation coefficient	Confidence Interval	chi^2^ test (hetrogeneity *p*-value)	I^2^	Test for interaction (*P*-value)
Sample size	> = 250	2	0.04	−0.09 to 0.17	0.09	64%	0.50
	< 250	4	0.34	−0.50 to 0.85	< 0.01	99%	
Score quality assessment	> = 6 (High quality)	2	−0.04	−0.14 to 0.07	0.80	0%	0.34
	< 6 (low quality)	4	0.37	−0.45 to 0.86	< 0.01	99%	
Design	Cross-sectional study	5	0.25	−0.43 to 0.75	< 0.01	99%	0.85
	Case control study	1	0.19	0.03 to 0.33	–	–	
Type of analysis	Bivariate analysis	3	0.39	−0.70 to 0.93	< 0.01	99%	0.61
	Multivariate analysis	3	0.08	−0.04 to 0.19	0.07	62%	
All study		6	0.24	−0.33 to 0.68	< 0.01	99%	–

## Discussion

The present systematic meta-analysis study was performed to identify the intermediate social determinants of health associated with sexual quality of life in women of reproductive age. Based on the question of this systematic and meta-analysis study, among the intermediate social determinants of health, variables of depression, body image, self-esteem, physical activity, sexual function, and marital relationship were found as factors associated with sexual quality of life. Nevertheless, in most of these associated factors, the intensity of the relationship with sexual quality of life was moderate. Meanwhile, for the relationship observed in the variables of body image and self-esteem, severe heterogeneity, and due to the limited number of studies in these variables, it was not possible to do subgroup analysis to identify the possible source of heterogeneity.

Based on the results of the present meta-analysis, it was found that from among the psychological factors of social determinants of health, depression had somehow a strong relationship with sexual quality of life in women. Depression affects the person’s emotions and can adversely influence different aspects of person’s life including the sexual dimension of life [15]. Studies have shown that depression is associated with diminished sexual drive and orgasm; and in a vicious cycle, the presence of sexual dysfunction itself increases the severity of depression in women [37, 38]. The results of a systematic review and meta-analysis to identify the risk factors associated with sexual function among reproductive age women by Alidost et al. indicated that one of the factors affecting the sexual function of women is depression [39]. The results of this study have been in line with the present study findings. Nevertheless, the main variable of their study was sexual functioning, but in the present study, it was sexual quality of life.

Another result of the present meta-analysis was positive association between quality of marital relation and sexual quality of life. Studies have reported that one of the factors affecting the quality of marital relationships is satisfaction with the sexual relations dimensions. Indeed, meeting the sexual expectations and needs of both partners has a positive effect on the quality of marital relationships [40, 41]. In a systematic review to determine the predictors of sexual satisfaction in women, it was indicated that the factor of marital relationships is one of the predictors of satisfaction in sexual relationships [42]. In addition, in the study by Roussin et al. done systematically to identify the factors affecting sexual quality of life, it was observed that the quality of marital relationships is a factor associated with sexual quality of life in women [43]. The results of this study also concur with the present study, though in that study the study population consisted of women suffering from cancer.

The relationship between sexual functioning and sexual quality of life has been another result obtained in the meta-analysis section of the present study. Women with better sexual functioning showed greater sexual quality of life. Sexual quality of life and functioning both are associated with the sexual dimension, and it seems that sexual functioning through sexual drive and its impact on personal, social, and familial relationships can influence the general quality of life and the sexual quality of life [44, 45].

Another result obtained in this study was association between body image plus self-confidence and sexual quality of life. Nevertheless, this result, due to limited number of primary studies (two studies) and high heterogeneity in both variables was an inconclusive result. It seems that positive body image by increasing sexual self-confidence in women can lead to satisfactory sexual experiences in sexual relationships [46]. Meanwhile, alteration of body image and reduction of self-confidence in women can also have a negative effect on the interactions between couples, which leads to diminished sexual quality of life [47]. In a review study to determine the factors associated with sexual functioning and satisfaction with sexual relationships, it was found that one of the factors affecting the sexual satisfaction and functioning is the body image of people [48]. This result was also found in the present study.

Another result obtained was association between the variable of physical activity and sexual quality of life. Nevertheless, this result considering the limited number of primary studies (two studies) was inconclusive. In a systematic study to evaluate the effect of physical activity on sexual functioning and sexual quality of life in postmenopausal women, it was found that the aerobic exercises were associated with discrepant results, while resistance exercises had no positive effect on the sexual quality of life of these women [49]. This result does not concur with our study. Nevertheless, this study had been done on postmenopausal women and considering the high degree of variety across the existent programs and the assessment methods employed, any conclusion in this regard should be drawn with caution.

The variable of duration of marriage as the second remaining in the present study had a weak relationship with high heterogeneity with sexual quality of life. With an increase in the duration of marriage, the sexual quality of life in women was better. The review study by Alidost et al. indicated that an increase in the duration of marriage is one of the risk factors of sexual dysfunction in women [39]. This has been in contradiction to our findings.

### Strengths and limitations of the study

One of the strengths of the present study was systematic assessment of all intermediate social determinants of health based on the WHO model and consideration of standard instruments in inclusion criteria. Regarding the limitations, given the limited number of primary studies in this domain, among the included studies, there were also women with a history of infertility, obesity, handicapped child, and women with endometriosis, and the methodological quality of most studies was low. Meanwhile, in the meta-analysis of the variables of body image and self-esteem, due to limited number of studies (two studies), it was not possible to perform subgroup analysis to identify the possible source of heterogeneity, evaluate publication bias, and perform sensitivity analysis. Thus, further primary studies are recommended to be performed in this area in order to achieve definite results in future. In the variable of relationships between couples, again in spite of performing subgroup analysis, the factors affecting reduction of heterogeneity was not identified. Eventually, it should be noted that the nature of cross-sectional studies is such that they cannot identify causal relationships.

## Conclusion

From among the intermediate social determinants of health, the variables of depression, body image, self-esteem, physical activity, sexual functioning, and quality of marital relation were associated with sexual quality of life. Nevertheless, this relationship was moderate in most of these factors. Identification of factors associated with sexual quality of life in women can be a forward step towards designing interventional studies and can help health service providers (nurses and midwives) to improve and promote sexual quality of life of women. In addition, it seems that paying attention to these factors by health planners of women sexual health is important. It is important to develop a comprehensive plan for promoting sexual quality of life of women by considering effective factors such as depression, body image, self-esteem, physical activity, sexual functioning, and quality of marital relation.

### Supplementary Information

**Additional file 1.** Search terms and strategies.

**Additional file 2.** Newcastle - Ottawa Quality Assessment Scale.

**Additional file 3.** Subgroup analysis (Forest plot).

## Data Availability

The data that support the findings of this study are available from the corresponding author upon reasonable request.

## Abbreviations

SQOL-F: Sexual Quality of Life-Female
WHO: Word Health Organization
LSQ: Life Style Questionnaire
BDI: Beck Depression Inventory
HADS: Hospital Anxiety and Depression Scale
PSS-14: Perceived Stress Scale
MPSS: Perceived Social Support
BIS: Body Image Scale
FGSIS-I: Female Genital Self-Image Scale
FSFI: Female Sexual Function Index
MINQ: Marital Intimacy Needs Questionnaire
DAS: Dyadic Adjustment Scale
