# Supplementary material for: Predictors of social intermediate factors associated with sexual quality of life of women: systematic review and meta-analysis
Source: BMC Womens Health. 2024 Jan 24;24:64. doi: 10.1186/s12905-024-02899-2 (PMC10809577; doi:10.1186/s12905-024-02899-2)
Supplement: Supplementary file 1 — Additional file 1. Search terms and strategies. [file 12905_2024_2899_MOESM1_ESM.doc]

**Search terms and strategies**

**Last searched April 10, 2021.**

**Compelete PubMed Syntax: 1259**

(("Social Determinants of Health"[Mesh] OR "Social Determinant of Health"[tiab] OR "Health Social Determinant"[tiab] OR "Health Risk Behaviors"[Mesh] OR (Behavior[tiab] AND "Health Risk"[tiab]) OR "Health Risk Behavior"[tiab] OR ("Risk Behavior"[tiab] AND Health[tiab]) OR "Risky Health Behavior"[tiab] OR (Behavior[tiab] AND "Risky Health"[tiab]) OR ("Health Behavior"[tiab] AND Risky[tiab]) OR (Behavior[tiab] AND Health[tiab]) OR "Health Behavior"[tiab] OR "Health-Related Behavior"[tiab] OR "Health Related Behavior"[tiab] OR (Behavior[tiab] AND Health-Related[tiab]) OR (Behavior[tiab] AND "Health Related"[tiab]) OR "Life Style"[Mesh] OR "Life Style"[tiab] OR Lifestyle[tiab] OR "Lifestyle Factor"[tiab] OR (Factor[tiab] AND Lifestyle[tiab]) OR "Lifestyle Factor"[tiab] OR (Lifestyle[tiab] AND Healthy[tiab]) OR "Healthy Lifestyle"[Mesh] OR "Healthy Life Style"[tiab] OR "Healthy Lifestyle"[tiab] OR ("Life Style"[tiab] AND Healthy[tiab]) OR "Social Support"[Mesh] OR "Social Support"[tiab] OR (Support[tiab] AND Social[tiab]) OR ("Social Support"[tiab] AND Online[tiab]) OR (Support[tiab] AND "Online Social"[tiab]) OR "Perceived Social Support"[tiab] OR ("Social Support"[tiab] AND Perceived[tiab]) OR (Support[tiab] AND "Perceived Social"[tiab]) OR "Mental Health"[Mesh] OR "Mental Health"[tiab] OR (Health[tiab] AND Mental[tiab]) OR "Mental Hygiene"[tiab] OR (Hygiene[tiab] AND Mental[tiab]) OR "Depression"[Mesh] OR Depression[tiab] OR "Depressive Symptom"[tiab] OR (Symptom[tiab] AND Depressive[tiab]) OR "Emotional Depression"[tiab] OR (Depression[tiab] AND Emotional[tiab]) OR "Anxiety"[Mesh] OR Anxiety[tiab] OR Angst[tiab] OR Nervousness[tiab] OR Hypervigilance[tiab] OR Anxiousness[tiab] OR "Social Anxiety"[tiab] OR (Anxiety[tiab] AND Social[tiab]) OR "Stress, Psychological"[Mesh] OR Stress[tiab] OR "Psychological Stress"[tiab] OR (Stresses[tiab] AND Psychological[tiab]) OR "Life Stress"[tiab] OR (Stress[tiab] AND Life[tiab]) OR (Stress[tiab] AND Psychologic[tiab]) OR "Psychologic Stress"[tiab] OR (Stressor[tiab] AND Psychological[tiab]) OR "Psychological Stressor"[tiab] OR "Health Services Accessibility"[Mesh] OR "Availability of Health Services"[tiab] OR "Health Services Availability"[tiab] OR "Accessibility of Health Services"[tiab] OR (Accessibility[tiab] AND "Health Services"[tiab]) OR "Access to Health Services"[tiab] OR "Access to Health Care"[tiab] OR "Health Services Geographic Accessibility"[tiab] OR "Program Accessibility"[tiab] OR (Accessibility[tiab] AND Program[tiab]) OR "Violence"[Mesh] OR Violence[tiab] OR Atrocities[tiab] OR "Structural Violence"[tiab] OR (Violence[tiab] AND Structural[tiab]) OR "Assaultive Behavior"[tiab] OR (Behavior[tiab] AND Assaultive[tiab]) OR "Domestic Violence"[tiab] OR (Violence[tiab] AND Domestic[tiab]) OR "Family Violence"[tiab] OR (Violence[tiab] AND Family[tiab]) OR "Body Image"[Mesh] OR "Body Image"[tiab] OR (Image[tiab] AND Body[tiab]) OR "Body Identity"[tiab] OR (Identity[tiab] AND Body[tiab]) OR "Body Representation"[tiab] OR (Representation[tiab] AND Body[tiab]) OR "Body Schema"[tiab] OR (Schema[tiab] AND Body[tiab]) OR "Self Concept"[Mesh] OR (Concept[tiab] AND Self[tiab]) OR SelfPerception[tiab] OR "Self Perception"[tiab] OR (Perception[tiab] AND Self[tiab]) OR "Self Confidence"[tiab] OR (Confidence[tiab] AND Self[tiab]) OR "Self Esteem"[tiab] OR (Esteem[tiab] AND Self[tiab]) OR "Exercise"[Mesh] OR Exercise[tiab] OR "Physical Activity"[tiab] OR (Activity[tiab] AND Physical[tiab]) OR "Smoking"[Mesh] OR Smoking[tiab] OR "Smoking Behavior"[tiab] OR (Behavior[tiab] AND Smoking[tiab]) OR "Smoking Habit"[tiab] OR (Habit[tiab] AND Smoking[tiab]) OR "Illicit Drugs"[Mesh] OR "Illicit Drug"[tiab] OR (Drug[tiab] AND Illicit[tiab]) OR "Illegal Drug"[tiab] OR (Drug[tiab] AND Illegal[tiab]) OR "Street Drug"[tiab] OR (Drug[tiab] AND Street[tiab]) OR "Recreational Drug"[tiab] OR (Drug[tiab] AND Recreational[tiab]) OR "Club Drug"[tiab] OR (Drug[tiab] AND Club[tiab]) OR (Behavior[tiab] AND Drug-Seeking[tiab]) OR (Behavior[tiab] AND DrugSeeking[tiab]) OR (Behavior[tiab] AND "Drug Seeking"[tiab]) OR "Drug Seeking Behavior"[tiab] OR “Marital satisfaction”[tiab] OR “Marital status”[Mesh] OR “Marital status”[tiab] OR (Status[tiab] AND Marital[tiab]) OR “[Marital Intimacy](https://healthjournal.arums.ac.ir/search.php?sid=1&slc_lang=en&key=Marital+Intimacy)”[tiab] OR “Marital Relationship”[tiab] OR (Relationship[tiab] AND Marital[tiab]) OR “Relationship Quality”[tiab] OR "Sexual function"[tiab] OR “FSFI”[tiab]) AND (("Sexual life"[tiab] OR "Quality of sexual life"[tiab] OR "Sexual quality"[tiab] OR "Sexual life quality"[tiab] OR "Sexual quality of life-female"[tiab] OR SQOL-F[tiab] AND 2010/01/01:2022/03/30[dp]))

**Compelete Scopus Syntax: 1877**

(TITLE-ABS-KEY("Social Determinants of Health") OR TITLE-ABS-KEY("Health Social Determinant") OR TITLE-ABS-KEY("Intermediate determinants of health") OR (TITLE-ABS-KEY(Behavior) AND TITLE-ABS-KEY("Health Risk")) OR TITLE-ABS-KEY("Health Risk Behavior") OR (TITLE-ABS-KEY("Risk Behavior") AND TITLE-ABS-KEY(Health)) OR TITLE-ABS-KEY"(Risky Health Behavior") OR (TITLE-ABS-KEY(Behavior) AND TITLE-ABS-KEY("Risky Health")) OR (TITLE-ABS-KEY("Health Behavior") AND TITLE-ABS-KEY(Risky)) OR (TITLE-ABS-KEY(Behavior) AND TITLE-ABS-KEY(Health)) OR TITLE-ABS-KEY("Health Behavior") OR TITLE-ABS-KEY("Health-Related Behavior") OR TITLE-ABS-KEY("Health Related Behavior") OR (TITLE-ABS-KEY(Behavior) AND TITLE-ABS-KEY(Health-Related)) OR (TITLE-ABS-KEY(Behavior) AND TITLE-ABS-KEY("Health Related")) OR TITLE-ABS-KEY("Life Style") OR TITLE-ABS-KEY(Lifestyle) OR TITLE-ABS-KEY("Lifestyle Factor") OR (TITLE-ABS-KEY(Factor) AND TITLE-ABS-KEY(Lifestyle)) OR TITLE-ABS-KEY("Lifestyle Factor") OR (TITLE-ABS-KEY(Lifestyle) AND TITLE-ABS-KEY(Healthy)) OR TITLE-ABS-KEY("Healthy Life Style") OR TITLE-ABS-KEY("Healthy Lifestyle") OR (TITLE-ABS-KEY("Life Style") AND TITLE-ABS-KEY(Healthy)) OR TITLE-ABS-KEY("Social Support") OR (TITLE-ABS-KEY(Support) AND TITLE-ABS-KEY(Social)) OR (TITLE-ABS-KEY("Social Support") AND TITLE-ABS-KEY(Online)) OR (TITLE-ABS-KEY(Support) AND TITLE-ABS-KEY("Online Social")) OR TITLE-ABS-KEY("Perceived Social Support") OR (TITLE-ABS-KEY("Social Support") AND TITLE-ABS-KEY(Perceived)) OR (TITLE-ABS-KEY(Support) AND TITLE-ABS-KEY("Perceived Social")) OR TITLE-ABS-KEY("Mental Health") OR (TITLE-ABS-KEY(Health) AND TITLE-ABS-KEY(Mental)) OR TITLE-ABS-KEY("Mental Hygiene") OR (TITLE-ABS-KEY(Hygiene) AND TITLE-ABS-KEY(Mental)) OR TITLE-ABS-KEY(Depression) OR TITLE-ABS-KEY("Depressive Symptom") OR (TITLE-ABS-KEY(Symptom) AND TITLE-ABS-KEY(Depressive)) OR TITLE-ABS-KEY("Emotional Depression") OR (TITLE-ABS-KEY(Depression) AND TITLE-ABS-KEY(Emotional)) OR TITLE-ABS-KEY(Anxiety) OR TITLE-ABS-KEY(Angst) OR TITLE-ABS-KEY(Nervousness) OR TITLE-ABS-KEY(Hypervigilance) OR TITLE-ABS-KEY(Anxiousness) OR TITLE-ABS-KEY("Social Anxiety") OR (TITLE-ABS-KEY(Anxiety) AND TITLE-ABS-KEY(Social)) OR TITLE-ABS-KEY(Stress) OR TITLE-ABS-KEY("Psychological Stress") OR (TITLE-ABS-KEY(Stresses) AND TITLE-ABS-KEY(Psychological)) OR TITLE-ABS-KEY("Life Stress") OR (TITLE-ABS-KEY(Stress) AND TITLE-ABS-KEY(Life)) OR (TITLE-ABS-KEY(Stress) AND TITLE-ABS-KEY(Psychologic)) OR TITLE-ABS-KEY("Psychologic Stress") OR (TITLE-ABS-KEY(Stressor) AND TITLE-ABS-KEY(Psychological)) OR TITLE-ABS-KEY("Psychological Stressor") OR TITLE-ABS-KEY("Availability of Health Services") OR TITLE-ABS-KEY("Health Services Availability") OR TITLE-ABS-KEY("Accessibility of Health Services") OR (TITLE-ABS-KEY(Accessibility) AND TITLE-ABS-KEY("Health Services")) OR TITLE-ABS-KEY("Access to Health Services") OR TITLE-ABS-KEY("Access to Health Care") OR TITLE-ABS-KEY("Health Services Geographic Accessibility") OR TITLE-ABS-KEY("Program Accessibility") OR (TITLE-ABS-KEY(Accessibility) AND TITLE-ABS-KEY(Program)) OR TITLE-ABS-KEY(Violence) OR TITLE-ABS-KEY(Atrocities) OR TITLE-ABS-KEY("Structural Violence") OR (TITLE-ABS-KEY(Violence) AND TITLE-ABS-KEY(Structural)) OR TITLE-ABS-KEY("Assaultive Behavior") OR (TITLE-ABS-KEY(Behavior) AND TITLE-ABS-KEY(Assaultive)) OR TITLE-ABS-KEY("Domestic Violence") OR (TITLE-ABS-KEY(Violence) AND TITLE-ABS-KEY(Domestic)) OR TITLE-ABS-KEY("Family Violence") OR (TITLE-ABS-KEY(Violence) AND TITLE-ABS-KEY(Family)) OR TITLE-ABS-KEY("Body Image") OR (TITLE-ABS-KEY(Image) AND TITLE-ABS-KEY(Body)) OR TITLE-ABS-KEY("Body Identity") OR (TITLE-ABS-KEY(Identity) AND TITLE-ABS-KEY(Body)) OR TITLE-ABS-KEY("Body Representation") OR (TITLE-ABS-KEY(Representation) AND TITLE-ABS-KEY(Body)) OR TITLE-ABS-KEY("Body Schema") OR (TITLE-ABS-KEY(Schema) AND TITLE-ABS-KEY(Body)) OR (TITLE-ABS-KEY(Concept) AND TITLE-ABS-KEY(Self)) OR TITLE-ABS-KEY(SelfPerception) OR TITLE-ABS-KEY("Self Perception") OR (TITLE-ABS-KEY(Perception) AND TITLE-ABS-KEY(Self)) OR TITLE-ABS-KEY("Self Confidence") OR (TITLE-ABS-KEY(Confidence) AND TITLE-ABS-KEY(Self)) OR TITLE-ABS-KEY("Self Esteem") OR (TITLE-ABS-KEY(Esteem) AND TITLE-ABS-KEY(Self)) OR TITLE-ABS-KEY(Exercise) OR TITLE-ABS-KEY("Physical Activity") OR (TITLE-ABS-KEY(Activity) AND TITLE-ABS-KEY(Physical)) OR TITLE-ABS-KEY(Smoking) OR TITLE-ABS-KEY("Smoking Behavior") OR (TITLE-ABS-KEY(Behavior) AND TITLE-ABS-KEY(Smoking)) OR TITLE-ABS-KEY("Smoking Habit") OR (TITLE-ABS-KEY(Habit) AND TITLE-ABS-KEY(Smoking)) OR TITLE-ABS-KEY("Illicit Drug") OR (TITLE-ABS-KEY(Drug) AND TITLE-ABS-KEY(Illicit)) OR TITLE-ABS-KEY("Illegal Drug") OR (TITLE-ABS-KEY(Drug) AND TITLE-ABS-KEY(Illegal)) OR TITLE-ABS-KEY("Street Drug") OR (TITLE-ABS-KEY(Drug) AND TITLE-ABS-KEY(Street)) OR TITLE-ABS-KEY("Recreational Drug") OR (TITLE-ABS-KEY(Drug) AND TITLE-ABS-KEY(Recreational)) OR TITLE-ABS-KEY("Club Drug") OR (TITLE-ABS-KEY(Drug) AND TITLE-ABS-KEY(Club)) OR (TITLE-ABS-KEY(Behavior) AND TITLE-ABS-KEY(Drug-Seeking)) OR (TITLE-ABS-KEY(Behavior) AND TITLE-ABS-KEY(DrugSeeking)) OR (TITLE-ABS-KEY(Behavior) AND TITLE-ABS-KEY("Drug Seeking")) OR TITLE-ABS-KEY("Drug Seeking Behavior") OR TITLE-ABS-KEY(“Marital satisfaction”) OR TITLE-ABS-KEY(“Marital status”) OR (TITLE-ABS-KEY(Status) AND TITLE-ABS-KEY(Marital)) OR TITLE-ABS-KEY(“[Marital Intimacy](https://healthjournal.arums.ac.ir/search.php?sid=1&slc_lang=en&key=Marital+Intimacy)”) OR TITLE-ABS-KEY(“Marital Relationship”) OR (TITLE-ABS-KEY(Relationship) AND TITLE-ABS-KEY(Marital)) OR TITLE-ABS-KEY(“Relationship Quality”) OR TITLE-ABS-KEY("sexual function") OR TITLE-ABS-KEY(“FSFI”) AND (TITLE-ABS-KEY("Sexual life") OR TITLE-ABS-KEY("Quality of sexual life") OR TITLE-ABS-KEY("Sexual quality") OR TITLE-ABS-KEY("Sexual life quality") OR TITLE-ABS-KEY("Sexual quality of life-female") OR TITLE-ABS-KEY(SQOL-F)) AND (PUBYEAR > 2009 AND PUBYEAR < 2022) OR PUBDATETXT(January 2022) OR PUBDATETXT(February 2022) OR PUBDATETXT(March 2022)

**Compelete Web of Sciences Syntax: 1394**

(TS=("Social Determinants of Health") OR TS=("Health Social Determinant") OR TS=("Intermediate determinants of health") OR (TS=(Behavior) AND TS=("Health Risk")) OR TS=("Health Risk Behavior") OR (TS=("Risk Behavior") AND TS=(Health)) OR TS=("Risky Health Behavior") OR (TS=(Behavior) AND TS=("Risky Health")) OR (TS=("Health Behavior") AND TS=(Risky)) OR (TS=(Behavior) AND TS=(Health)) OR TS=("Health Behavior") OR TS=("Health-Related Behavior") OR TS=("Health Related Behavior") OR (TS=(Behavior) AND TS=(Health-Related)) OR (TS=(Behavior) AND TS=("Health Related")) OR TS=("Life Style") OR TS=(Lifestyle) OR TS=("Lifestyle Factor") OR (TS=(Factor) AND TS=(Lifestyle)) OR TS=("Lifestyle Factor") OR (TS=(Lifestyle) AND TS=(Healthy)) OR TS=("Healthy Life Style") OR TS=("Healthy Lifestyle") OR (TS=("Life Style") AND TS=(Healthy)) OR TS=("Social Support") OR (TS=(Support) AND TS=(Social)) OR (TS=("Social Support") AND TS=(Online)) OR (TS=(Support) AND TS=("Online Social")) OR TS=("Perceived Social Support") OR (TS=("Social Support") AND TS=(Perceived)) OR (TS=(Support) AND TS=("Perceived Social")) OR TS=("Mental Health") OR (TS=(Health) AND TS=(Mental)) OR TS=("Mental Hygiene") OR (TS=(Hygiene) AND TS=(Mental)) OR TS=(Depression) OR TS=("Depressive Symptom") OR (TS=(Symptom) AND TS=(Depressive)) OR TS=("Emotional Depression") OR (TS=(Depression) AND TS=(Emotional)) OR TS=(Anxiety) OR TS=(Angst) OR TS=(Nervousness) OR TS=(Hypervigilance) OR TS=(Anxiousness) OR TS=("Social Anxiety") OR (TS=(Anxiety) AND TS=(Social)) OR TS=(Stress) OR TS=("Psychological Stress") OR (TS=(Stresses) AND TS=(Psychological)) OR TS=("Life Stress") OR (TS=(Stress) AND TS=(Life)) OR (TS=(Stress) AND TS=(Psychologic)) OR TS=("Psychologic Stress") OR (TS=(Stressor) AND TS=(Psychological)) OR TS=("Psychological Stressor") OR TS=("Availability of Health Services") OR TS=("Health Services Availability") OR TS=("Accessibility of Health Services") OR (TS=(Accessibility) AND TS=("Health Services")) OR TS=("Access to Health Services") OR TS=("Access to Health Care") OR TS=("Health Services Geographic Accessibility") OR TS=("Program Accessibility") OR (TS=(Accessibility) AND TS=(Program)) OR TS=(Violence) OR TS=(Atrocities) OR TS=("Structural Violence") OR (TS=(Violence) AND TS=(Structural)) OR TS=("Assaultive Behavior") OR (TS=(Behavior) AND TS=(Assaultive)) OR TS=("Domestic Violence") OR (TS=(Violence) AND TS=(Domestic)) OR TS=("Family Violence") OR (TS=(Violence) AND TS=(Family)) OR TS=("Body Image") OR (TS=(Image) AND TS=(Body)) OR TS=("Body Identity") OR (TS=(Identity) AND TS=(Body)) OR TS=("Body Representation") OR (TS=(Representation) AND TS=(Body)) OR TS=("Body Schema") OR (TS=(Schema) AND TS=(Body)) OR (TS=(Concept) AND TS=(Self)) OR TS=(Self-perception) OR TS=("Self Perception") OR (TS=(Perception) AND TS=(Self)) OR TS=("Self Confidence") OR (TS=(Confidence) AND TS=(Self)) OR TS=("Self Esteem") OR (TS=(Esteem) AND TS=(Self)) OR TS=(Exercise) OR TS=("Physical Activity") OR (TS=(Activity) AND TS=(Physical)) OR TS=(Smoking) OR TS=("Smoking Behavior") OR (TS=(Behavior) AND TS=(Smoking)) OR TS=("Smoking Habit") OR (TS=(Habit) AND TS=(Smoking)) OR TS=("Illicit Drug") OR (TS=(Drug) AND TS=(Illicit)) OR TS=("Illegal Drug") OR (TS=(Drug) AND TS=(Illegal)) OR TS=("Street Drug") OR (TS=(Drug) AND TS=(Street)) OR TS=("Recreational Drug") OR (TS=(Drug) AND TS=(Recreational)) OR TS=("Club Drug") OR (TS=(Drug) AND TS=(Club)) OR (TS=(Behavior) AND TS=(Drug-Seeking)) OR (TS=(Behavior) AND TS=(DrugSeeking)) OR (TS=(Behavior) AND TS=("Drug Seeking")) OR TS=("Drug Seeking Behavior") OR TS=(“Marital satisfaction”) OR TS=(“Marital status”) OR (TS=(Status) AND TS=(Marital)) OR TS=(“[Marital Intimacy](https://healthjournal.arums.ac.ir/search.php?sid=1&slc_lang=en&key=Marital+Intimacy)”) OR TS=(“Marital Relationship”) OR (TS=(Relationship) AND TS=(Marital)) OR TS=(“Relationship Quality”) OR TS=("sexual function") OR TS=(“FSFI”)) AND (TS=("Sexual life") OR TS=("Quality of sexual life") OR TS=("Sexual quality") OR TS=("Sexual life quality") OR TS=("Sexual quality of life-female") OR TS=(SQOL-F)) AND (PY=2010-2022)

**Compelete Embase Syntax: 1358**

("Social Determinants of Health":ti,ab,kw OR "Health Social Determinant":ti,ab,kw OR "Intermediate determinants of health":ti,ab,kw OR (Behavior:ti,ab,kw AND "Health Risk":ti,ab,kw) OR “Health Behavior”/exp OR "Health Risk Behavior":ti,ab,kw OR ("Risk Behavior":ti,ab,kw AND Health:ti,ab,kw) OR "Risky Health Behavior":ti,ab,kw OR (Behavior:ti,ab,kw AND "Risky Health":ti,ab,kw) OR ("Health Behavior":ti,ab,kw AND Risky:ti,ab,kw) OR (Behavior:ti,ab,kw AND Health:ti,ab,kw) OR "Health Behavior":ti,ab,kw OR "Health-Related Behavior":ti,ab,kw OR "Health Related Behavior":ti,ab,kw OR (Behavior:ti,ab,kw AND Health-Related:ti,ab,kw) OR (Behavior:ti,ab,kw AND "Health Related":ti,ab,kw) OR "Life Style":ti,ab,kw OR Lifestyle:ti,ab,kw OR "Lifestyle Factor":ti,ab,kw OR (Factor:ti,ab,kw AND Lifestyle:ti,ab,kw) OR "Lifestyle Factor":ti,ab,kw OR (Lifestyle:ti,ab,kw AND Healthy:ti,ab,kw) OR "Healthy Life Style":ti,ab,kw OR “Healthy lifestyle”/exp OR "Healthy Lifestyle":ti,ab,kw OR ("Life Style":ti,ab,kw AND Healthy:ti,ab,kw) OR "Social Support"/exp OR "Social Support":ti,ab,kw OR (Support:ti,ab,kw AND Social:ti,ab,kw) OR ("Social Support":ti,ab,kw AND Online:ti,ab,kw) OR (Support:ti,ab,kw AND "Online Social":ti,ab,kw) OR "Perceived Social Support":ti,ab,kw OR ("Social Support":ti,ab,kw AND Perceived:ti,ab,kw) OR (Support:ti,ab,kw AND "Perceived Social":ti,ab,kw) OR "Mental Health"/exp OR "Mental Health":ti,ab,kw OR (Health:ti,ab,kw AND Mental:ti,ab,kw) OR "Mental Hygiene":ti,ab,kw OR (Hygiene:ti,ab,kw AND Mental:ti,ab,kw) OR Depression/exp OR Depression:ti,ab,kw OR "Depressive Symptom":ti,ab,kw OR (Symptom:ti,ab,kw AND Depressive:ti,ab,kw) OR "Emotional Depression":ti,ab,kw OR (Depression:ti,ab,kw AND Emotional:ti,ab,kw) OR Anxiety/exp OR Anxiety:ti,ab,kw OR Angst:ti,ab,kw OR Nervousness:ti,ab,kw OR Hypervigilance:ti,ab,kw OR Anxiousness:ti,ab,kw OR "Social Anxiety":ti,ab,kw OR (Anxiety:ti,ab,kw AND Social:ti,ab,kw) OR Stress:ti,ab,kw OR "Psychological Stress"/exp OR "Psychological Stress":ti,ab,kw OR (Stresses:ti,ab,kw AND Psychological:ti,ab,kw) OR "Life Stress":ti,ab,kw OR (Stress:ti,ab,kw AND Life:ti,ab,kw) OR (Stress:ti,ab,kw AND Psychologic:ti,ab,kw) OR "Psychologic Stress":ti,ab,kw OR (Stressor:ti,ab,kw AND Psychological:ti,ab,kw) OR "Psychological Stressor":ti,ab,kw OR "Availability of Health Services":ti,ab,kw OR "Health Services Availability":ti,ab,kw OR "Accessibility of Health Services":ti,ab,kw OR (Accessibility:ti,ab,kw AND "Health Services":ti,ab,kw) OR "Access to Health Services":ti,ab,kw OR "Access to Health Care":ti,ab,kw OR "Health Services Geographic Accessibility":ti,ab,kw OR "Program Accessibility":ti,ab,kw OR (Accessibility:ti,ab,kw AND Program:ti,ab,kw) OR Violence:ti,ab,kw OR Atrocities:ti,ab,kw OR "Structural Violence":ti,ab,kw OR Violence/exp OR (Violence:ti,ab,kw AND Structural:ti,ab,kw) OR "Assaultive Behavior":ti,ab,kw OR (Behavior:ti,ab,kw AND Assaultive:ti,ab,kw) OR "Domestic Violence":ti,ab,kw OR (Violence:ti,ab,kw AND Domestic:ti,ab,kw) OR "Family Violence":ti,ab,kw OR (Violence:ti,ab,kw AND Family:ti,ab,kw) OR "Body Image"/exp OR "Body Image":ti,ab,kw OR (Image:ti,ab,kw AND Body:ti,ab,kw) OR "Body Identity":ti,ab,kw OR (Identity:ti,ab,kw AND Body:ti,ab,kw) OR "Body Representation":ti,ab,kw OR (Representation:ti,ab,kw AND Body:ti,ab,kw) OR "Body Schema":ti,ab,kw OR (Schema:ti,ab,kw AND Body:ti,ab,kw) OR (Concept:ti,ab,kw AND Self:ti,ab,kw) OR SelfPerception:ti,ab,kw OR "Self Perception":ti,ab,kw OR (Perception:ti,ab,kw AND Self:ti,ab,kw) OR "Self Confidence":ti,ab,kw OR (Confidence:ti,ab,kw AND Self:ti,ab,kw) OR "Self Esteem"/exp OR "Self Esteem":ti,ab,kw OR (Esteem:ti,ab,kw AND Self:ti,ab,kw) OR Exercise:ti,ab,kw OR "Physical Activity"/exp OR "Physical Activity":ti,ab,kw OR (Activity:ti,ab,kw AND Physical:ti,ab,kw) OR Smoking:ti,ab,kw OR "Smoking Behavior":ti,ab,kw OR (Behavior:ti,ab,kw AND Smoking:ti,ab,kw) OR "Smoking Habit":ti,ab,kw OR (Habit:ti,ab,kw AND Smoking:ti,ab,kw) OR "Illicit Drug"/exp OR "Illicit Drug":ti,ab,kw OR (Drug:ti,ab,kw AND Illicit:ti,ab,kw) OR "Illegal Drug":ti,ab,kw OR (Drug:ti,ab,kw AND Illegal:ti,ab,kw) OR "Street Drug":ti,ab,kw OR (Drug:ti,ab,kw AND Street:ti,ab,kw) OR "Recreational Drug":ti,ab,kw OR (Drug:ti,ab,kw AND Recreational:ti,ab,kw) OR "Club Drug":ti,ab,kw OR (Drug:ti,ab,kw AND Club:ti,ab,kw) OR (Behavior:ti,ab,kw AND Drug-Seeking:ti,ab,kw) OR (Behavior:ti,ab,kw AND DrugSeeking:ti,ab,kw) OR (Behavior:ti,ab,kw AND "Drug Seeking":ti,ab,kw) OR "Drug Seeking Behavior":ti,ab,kw OR “Marital satisfaction”:ti,ab,kw OR “Marital status”:ti,ab,kw OR (Status:ti,ab,kw AND Marital:ti,ab,kw) OR “[Marital Intimacy](https://healthjournal.arums.ac.ir/search.php?sid=1&slc_lang=en&key=Marital+Intimacy)”:ti,ab,kw OR “Marital Relationship”:ti,ab,kw OR (Relationship:ti,ab,kw AND Marital:ti,ab,kw) OR “Relationship Quality”:ti,ab,kw OR "sexual function":ti,ab,kw OR “FSFI”:ti,ab,kw) AND (("Sexual life":ti,ab,kw OR "Quality of sexual life":ti,ab,kw OR "Sexual quality":ti,ab,kw OR "Sexual life quality":ti,ab,kw OR "Sexual quality of life-female":ti,ab,kw OR SQOL-F:ti,ab,kw AND [2010-2022]/py))

**Compelete Chochrane Syntax: 299**

("Social Determinants of Health" OR "Health Social Determinant" OR "Intermediate determinants of health" OR (Behavior AND "Health Risk") OR "Health Risk Behavior" OR ("Risk Behavior" AND Health) OR "Risky Health Behavior" OR (Behavior AND "Risky Health") OR ("Health Behavior" AND Risky) OR (Behavior AND Health) OR "Health Behavior" OR "Health-Related Behavior" OR "Health Related Behavior" OR (Behavior AND Health-Related) OR (Behavior AND "Health Related") OR "Life Style" OR Lifestyle OR "Lifestyle Factor" OR (Factor AND Lifestyle) OR "Lifestyle Factor" OR (Lifestyle AND Healthy) OR "Healthy Lifestyle"[Mesh] OR "Healthy Life Style" OR "Healthy Lifestyle" OR ("Life Style" AND Healthy) OR "Social Support" OR (Support AND Social) OR ("Social Support" AND Online) OR (Support AND "Online Social") OR "Perceived Social Support" OR ("Social Support" AND Perceived) OR (Support AND "Perceived Social") OR "Mental Health" OR (Health AND Mental) OR "Mental Hygiene" OR (Hygiene AND Mental) OR Depression OR "Depressive Symptom" OR (Symptom AND Depressive) OR "Emotional Depression" OR (Depression AND Emotional) OR Anxiety OR Angst OR Nervousness OR Hypervigilance OR Anxiousness OR "Social Anxiety" OR (Anxiety AND Social) OR Stress OR "Psychological Stress" OR (Stresses AND Psychological) OR "Life Stress" OR (Stress AND Life) OR (Stress AND Psychologic) OR "Psychologic Stress" OR (Stressor AND Psychological) OR "Psychological Stressor" OR "Availability of Health Services" OR "Health Services Availability" OR "Accessibility of Health Services" OR (Accessibility AND "Health Services") OR "Access to Health Services" OR "Access to Health Care" OR "Health Services Geographic Accessibility" OR "Program Accessibility" OR (Accessibility AND Program) OR Violence OR Atrocities OR "Structural Violence" OR (Violence AND Structural) OR "Assaultive Behavior" OR (Behavior AND Assaultive) OR "Domestic Violence" OR (Violence AND Domestic) OR "Family Violence" OR (Violence AND Family) OR "Body Image" OR (Image AND Body) OR "Body Identity" OR (Identity AND Body) OR "Body Representation" OR (Representation AND Body) OR "Body Schema" OR (Schema AND Body) OR (Concept AND Self) OR SelfPerception OR "Self Perception" OR (Perception AND Self) OR "Self Confidence" OR (Confidence AND Self) OR "Self Esteem" OR (Esteem AND Self) OR Exercise OR "Physical Activity" OR (Activity AND Physical) OR Smoking OR "Smoking Behavior" OR (Behavior AND Smoking) OR "Smoking Habit" OR (Habit AND Smoking) OR "Illicit Drug" OR (Drug AND Illicit) OR "Illegal Drug" OR (Drug AND Illegal) OR "Street Drug" OR (Drug AND Street) OR "Recreational Drug" OR (Drug AND Recreational) OR "Club Drug" OR (Drug AND Club) OR (Behavior AND Drug-Seeking) OR (Behavior AND DrugSeeking) OR (Behavior AND "Drug Seeking") OR "Drug Seeking Behavior" OR “Marital satisfaction” OR “Marital status” OR (Status AND Marital) OR “[Marital Intimacy](https://healthjournal.arums.ac.ir/search.php?sid=1&slc_lang=en&key=Marital+Intimacy)” OR “Marital Relationship” OR (Relationship AND Marital) OR “Relationship Quality” OR "sexual function" OR “FSFI”) AND ("Sexual life" OR "Quality of sexual life" OR "Sexual quality" OR "Sexual life quality" OR "Sexual quality of life-female" OR SQOL-F) AND (2010:2022)

**Compelete ProQuest Syntax: 205**

(AB,TI("Social Determinants of Health") OR AB,TI("Health Social Determinant") OR AB,TI("Intermediate determinants of health") OR (AB,TI(Behavior) AND AB,TI("Health Risk")) OR AB,TI("Health Risk Behavior") OR (AB,TI("Risk Behavior") AND AB,TI(Health)) OR AB,TI(“Risky Health Behavior") OR (AB,TI(Behavior) AND AB,TI("Risky Health")) OR (AB,TI("Health Behavior") AND AB,TI(Risky)) OR (AB,TI(Behavior) AND AB,TI(Health)) OR AB,TI("Health Behavior") OR AB,TI("Health-Related Behavior") OR AB,TI("Health Related Behavior") OR (AB,TI(Behavior) AND AB,TI(Health-Related)) OR (AB,TI(Behavior) AND AB,TI("Health Related")) OR AB,TI("Life Style") OR AB,TI(Lifestyle) OR AB,TI("Lifestyle Factor") OR (AB,TI(Factor) AND AB,TI(Lifestyle)) OR AB,TI("Lifestyle Factor") OR (AB,TI(Lifestyle) AND AB,TI(Healthy)) OR AB,TI("Healthy Life Style") OR AB,TI("Healthy Lifestyle") OR (AB,TI("Life Style") AND AB,TI(Healthy)) OR AB,TI("Social Support") OR (AB,TI(Support) AND AB,TI(Social)) OR (AB,TI("Social Support") AND AB,TI(Online)) OR (AB,TI(Support) AND AB,TI("Online Social")) OR AB,TI("Perceived Social Support") OR (AB,TI("Social Support") AND AB,TI(Perceived)) OR (AB,TI(Support) AND AB,TI("Perceived Social")) OR AB,TI("Mental Health") OR (AB,TI(Health) AND AB,TI(Mental)) OR AB,TI("Mental Hygiene") OR (AB,TI(Hygiene) AND AB,TI(Mental)) OR AB,TI(Depression) OR AB,TI("Depressive Symptom") OR (AB,TI(Symptom) AND AB,TI(Depressive)) OR AB,TI("Emotional Depression") OR (AB,TI(Depression) AND AB,TI(Emotional)) OR AB,TI(Anxiety) OR AB,TI(Angst) OR AB,TI(Nervousness) OR AB,TI(Hypervigilance) OR AB,TI(Anxiousness) OR AB,TI("Social Anxiety") OR (AB,TI(Anxiety) AND AB,TI(Social)) OR AB,TI(Stress) OR AB,TI("Psychological Stress") OR (AB,TI(Stresses) AND AB,TI(Psychological)) OR AB,TI("Life Stress") OR (AB,TI(Stress) AND AB,TI(Life)) OR (AB,TI(Stress) AND AB,TI(Psychologic)) OR AB,TI("Psychologic Stress") OR (AB,TI(Stressor) AND AB,TI(Psychological)) OR AB,TI("Psychological Stressor") OR AB,TI("Availability of Health Services") OR AB,TI("Health Services Availability") OR AB,TI("Accessibility of Health Services") OR (AB,TI(Accessibility) AND AB,TI("Health Services")) OR AB,TI("Access to Health Services") OR AB,TI("Access to Health Care") OR AB,TI("Health Services Geographic Accessibility") OR AB,TI("Program Accessibility") OR (AB,TI(Accessibility) AND AB,TI(Program)) OR AB,TI(Violence) OR AB,TI(Atrocities) OR AB,TI("Structural Violence") OR (AB,TI(Violence) AND AB,TI(Structural)) OR AB,TI("Assaultive Behavior") OR (AB,TI(Behavior) AND AB,TI(Assaultive)) OR AB,TI("Domestic Violence") OR (AB,TI(Violence) AND AB,TI(Domestic)) OR AB,TI("Family Violence") OR (AB,TI(Violence) AND AB,TI(Family)) OR AB,TI("Body Image") OR (AB,TI(Image) AND AB,TI(Body)) OR AB,TI("Body Identity") OR (AB,TI(Identity) AND AB,TI(Body)) OR AB,TI("Body Representation") OR (AB,TI(Representation) AND AB,TI(Body)) OR AB,TI("Body Schema") OR (AB,TI(Schema) AND AB,TI(Body)) OR (AB,TI(Concept) AND AB,TI(Self)) OR AB,TI(SelfPerception) OR AB,TI("Self Perception") OR (AB,TI(Perception) AND AB,TI(Self)) OR AB,TI("Self Confidence") OR (AB,TI(Confidence) AND AB,TI(Self)) OR AB,TI("Self Esteem") OR (AB,TI(Esteem) AND AB,TI(Self)) OR AB,TI(Exercise) OR AB,TI("Physical Activity") OR (AB,TI(Activity) AND AB,TI(Physical)) OR AB,TI(Smoking) OR AB,TI("Smoking Behavior") OR (AB,TI(Behavior) AND AB,TI(Smoking)) OR AB,TI("Smoking Habit") OR (AB,TI(Habit) AND AB,TI(Smoking)) OR AB,TI("Illicit Drug") OR (AB,TI(Drug) AND AB,TI(Illicit)) OR AB,TI("Illegal Drug") OR (AB,TI(Drug) AND AB,TI(Illegal)) OR AB,TI("Street Drug") OR (AB,TI(Drug) AND AB,TI(Street)) OR AB,TI("Recreational Drug") OR (AB,TI(Drug) AND AB,TI(Recreational)) OR AB,TI("Club Drug") OR (AB,TI(Drug) AND AB,TI(Club)) OR (AB,TI(Behavior) AND AB,TI(Drug-Seeking)) OR (AB,TI(Behavior) AND AB,TI(DrugSeeking)) OR (AB,TI(Behavior) AND AB,TI("Drug Seeking")) OR AB,TI("Drug Seeking Behavior") OR AB,TI(“Marital satisfaction”) OR AB,TI(“Marital status”) OR (AB,TI(Status) AND AB,TI(Marital)) OR AB,TI(“[Marital Intimacy](https://healthjournal.arums.ac.ir/search.php?sid=1&slc_lang=en&key=Marital+Intimacy)”) OR AB,TI(“Marital Relationship”) OR (AB,TI(Relationship) AND AB,TI(Marital)) OR AB,TI(“Relationship Quality”) OR AB,TI("sexual function") OR AB,TI(“FSFI”)) AND (AB,TI("Sexual life") OR AB,TI("Quality of sexual life") OR AB,TI("Sexual quality") OR AB,TI("Sexual life quality") OR AB,TI("Sexual quality of life-female") OR AB,TI(SQOL-F)) AND PD(20100130-20220330)
