# Supplementary material for: Predictors of social intermediate factors associated with sexual quality of life of women: systematic review and meta-analysis
Source: BMC Womens Health. 2024 Jan 24;24:64. doi: 10.1186/s12905-024-02899-2 (PMC10809577; doi:10.1186/s12905-024-02899-2)
Supplement: Supplementary file 2 — Additional file 2. Newcastle - Ottawa Quality Assessment Scale. [file 12905_2024_2899_MOESM2_ESM.doc]

**Newcastle - Ottawa Quality Assessment Scale**

**I) Cross Sectional Studies**

**Selection: (Maximum 5 stars)**

**1) Representativeness of the sample:**

a) Truly representative of the average in the target population. * (all subjects or random sampling)

b) Somewhat representative of the average in the target population. * (nonrandom sampling)

c) Selected group of users.

d) No description of the sampling strategy.

**2) Sample size:**

a) Justified and satisfactory. *

b) Not justified.

**3) Non-respondents:**

a) Comparability between respondents and non-respondents characteristics is established, and the response rate is satisfactory. *

b) The response rate is unsatisfactory, or the comparability between respondents and non-respondents is unsatisfactory.

c) No description of the response rate or the characteristics of the responders and the non-responders.

**4) Ascertainment of the exposure (risk factor):**

a) Validated measurement tool. **

b) Non-validated measurement tool, but the tool is available or described.*

c) No description of the measurement tool.

**Comparability: (Maximum 2 stars)**

**1) The subjects in different outcome groups are comparable, based on the study design or analysis. Confounding factors are controlled.**

a) The study controls for the most important factor (select one). *

b) The study control for any additional factor. *

**Outcome: (Maximum 3 stars)**

**1) Assessment of the outcome:**

a) Independent blind assessment. **

b) Record linkage. **

c) Self report. *

d) No description.

**2) Statistical test:**

a) The statistical test used to analyze the data is clearly described and appropriate, and the measurement of the association is presented, including confidence intervals and the probability level (p value). *

b) The statistical test is not appropriate, not described or incomplete.

**II) Case-Control Studies**

**Selection**

**1) Is the case definition adequate?**

a) yes, with independent validation *

b) yes, e.g., record linkage or based on self reports

c) no description

**2) Representativeness of the cases**

a) consecutive or obviously representative series of cases *

b) potential for selection biases or not stated

**3) Selection of Controls**

a) community controls *

b) hospital controls

c) no description

**4) Definition of Controls**

a) no history of disease (endpoint) *

b) no description of source Comparability

**Comparability**

**1) Comparability of cases and controls on the basis of the design or analysis**

a) study controls for _______________ (Select the most important factor.) *

b) study controls for any additional factor * (This criteria could be modified to indicate specific control for a second important factor.)

**Exposure**

**1) Ascertainment of exposure**

a) secure record (eg surgical records) *

b) structured interview where blind to case/control status *

c) interview not blinded to case/control status

d) written self report or medical record only

e) no description

**2) Same method of ascertainment for cases and controls**

a) yes *

b) no

**3) Non-Response rate**

a) same rate for both groups *

b) non respondents described

c) rate different and no designation

**III) Cohort Studies**

**Selection**

**1) Representativeness of the exposed cohort**

a) Truly representative (one star)

b) Somewhat representative (one star)

c) Selected group

d) No description of the derivation of the cohort

**2) Selection of the non-exposed cohort**

a) Drawn from the same community as the exposed cohort (one star)

b) Drawn from a different source

c) No description of the derivation of the non exposed cohort

**3) Ascertainment of exposure**

a) Secure record (e.g., surgical record) (one star)

b) Structured interview (one star)

c) Written self report

d) No description

e) Other

**4) Demonstration that outcome of interest was not present at start of study**

a) Yes (one star)

b) No Comparability

**Comparability**

**1) Comparability of cohorts on the basis of the design or analysis controlled for confounders**

a) The study controls for age, sex and marital status (one star)

b) Study controls for other factors (list) _________________________________ (one star)

c) Cohorts are not comparable on the basis of the design or analysis controlled for confounders **Outcome**

**1) Assessment of outcome**

a) Independent blind assessment (one star)

b) Record linkage (one star)

c) Self report

d) No description

e) Other

**2) Was follow-up long enough for outcomes to occur**

a) Yes (one star)

b) No Indicate the median duration of follow-up and a brief rationale for the assessment above:____________________

**3) Adequacy of follow-up of cohorts**

a) Complete follow up- all subject accounted for (one star)

b) Subjects lost to follow up unlikely to introduce bias- number lost less than or equal to 20% or description of those lost suggested no different from those followed. (one star)

c) Follow up rate less than 80% and no description of those lost

d) No statement

| **Results Of Newcastle-Ottawa Risk Of Bias Assessment (Cross-sectional study)** | | | | | | | | |
| --- | --- | --- | --- | --- | --- | --- | --- | --- |
| **Study ID** | Representativeness | Sample size | (Non-respondents) | (Ascertainment of the exposure risk factor) | (Confounding factors are controlled (based on the study design or analysis) | (Assessment of the outcome)me | Statistics | Total |
| Sheikhan et al., 2019  2019 | * | * | * | ** | ** | - | * | 8 |
| Türkben Polat and Kaplan Serin  2021 | - | * | - | ** | * | - | * | 6 |
| Samimi et al.,  2016 | - | - | - | ** | * | - | * | 4 |
| Tugut et al.,  2021 | - | - | - | ** | * | - | * | 4 |
| Tav et al.,  2018 | * | - | - | ** | * | - | * | 5 |
| Taskin Yilmaz et al., 2019 | * | - | - | ** | - | - | * | 4 |
| Shahraki et al.,  2016 | * | * | - | ** | * | - | * | 6 |
| Haghi et al.,  2018 | * | * | * | ** | - | * | * | 7 |
| Velayati et al.,  2021 | - | * | * | ** | * | * | * | 7 |
| Panahi et al.,  2021 | * | * | - | ** | - | - | * | 5 |
| Alcalde et al.,  2021 | - | * | - | ** | * | - | * | 5 |
| Yoksekol et al., 2021 | - | * | - | ** | * | - | * | 5 |

| **Results Of Newcastle-Ottawa Risk Of Bias Assessment (Case-control)** | | | | | | | | |  |
| --- | --- | --- | --- | --- | --- | --- | --- | --- | --- |
| **Study ID** | Case definition adequate | Representativeness of the cases | Selection of Controls | Definition of Controls | Comparability of cases and controls on the basis of the design or analysis | Ascertainment of exposure | Same method of ascertainment for cases and controls | Non-Response rate | Total score |
| Telli et al.,  2020 | - | - | - | * | - | * | * | * | 4 |
| Eftekhar et al.,  2021 | * | * | - | - | * | * | * | - | 5 |

| **Results Of Newcastle-Ottawa Risk Of Bias Assessment (Cohort study)** | | | | | | | | |  |
| --- | --- | --- | --- | --- | --- | --- | --- | --- | --- |
| **Study ID** | Representativeness of the exposed cohort | Selection of the non-exposed cohort | Ascertainment of exposure | Demonstration that outcome of interest was not present at start of study | Comparability of cohorts on the basis of the design or analysis controlled for confounders | Assessment of outcome | Was follow-up long enough for outcomes to occur | Adequacy of follow-up of cohorts | Total score |
| Brunault et al.,  2015 | - | - | * | - | * | - | * | * | 4 |
