# Supplementary material for: Predictors of social intermediate factors associated with sexual quality of life of women: systematic review and meta-analysis
Source: BMC Womens Health. 2024 Jan 24;24:64. doi: 10.1186/s12905-024-02899-2 (PMC10809577; doi:10.1186/s12905-024-02899-2)
Supplement: Supplementary file 3 — Additional file 3. Subgroup analysis (Forest plot). [file 12905_2024_2899_MOESM3_ESM.doc]

**Subgroup analysis (Forest plot)**


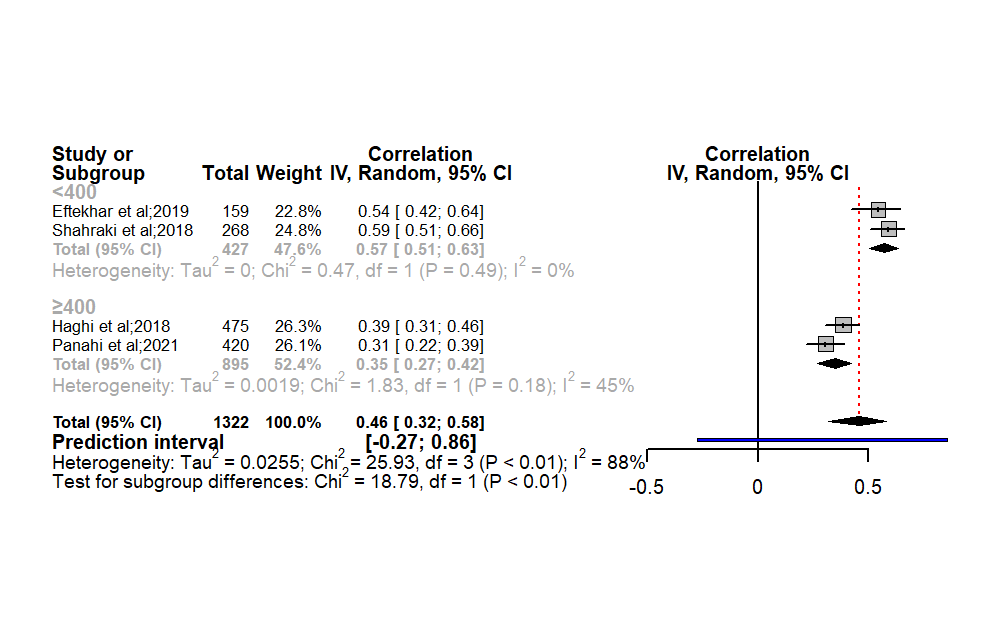


Figure S1: Subgroup analysis correlation coefficient with 95% confidence interval between sexual function and quality of sexual life by sample size


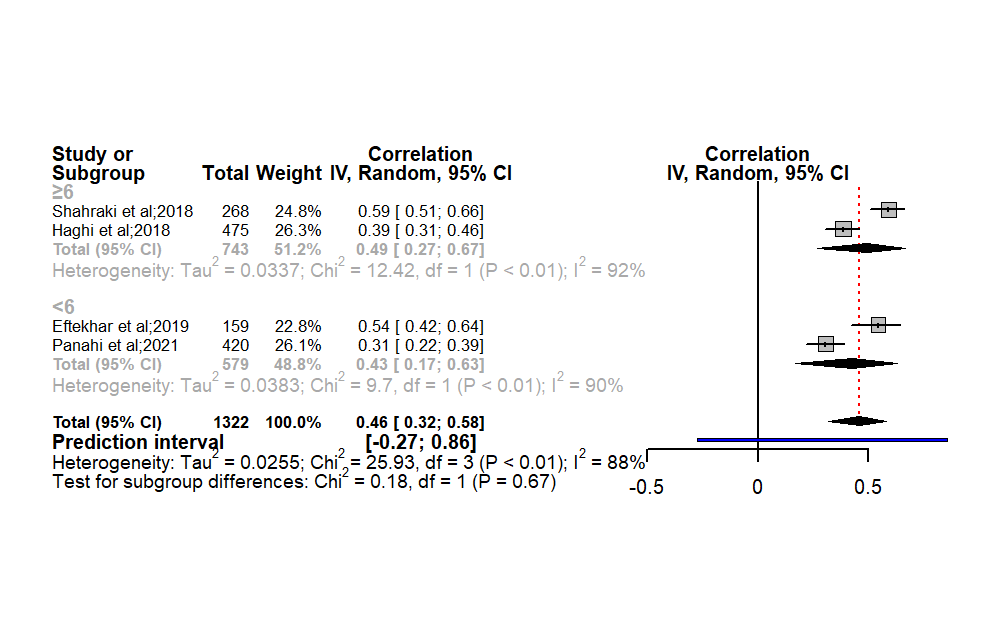


Figure S2: Subgroup analysis correlation coefficient with 95% confidence interval between sexual function and quality of sexual life by quality assessment


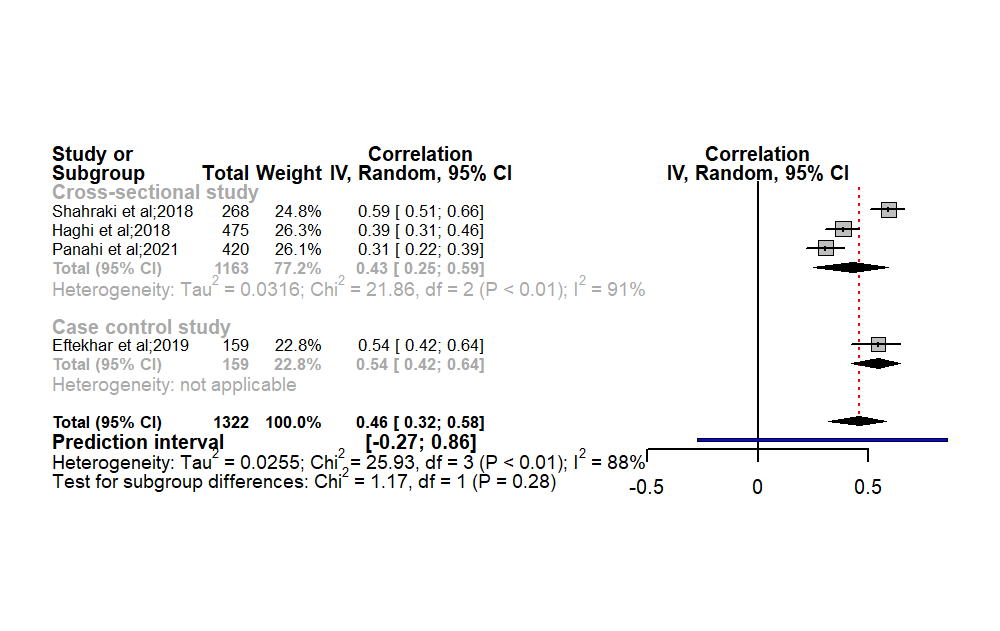


Figure S3: Subgroup analysis correlation coefficient with 95% confidence interval between sexual function and quality of sexual life by study design


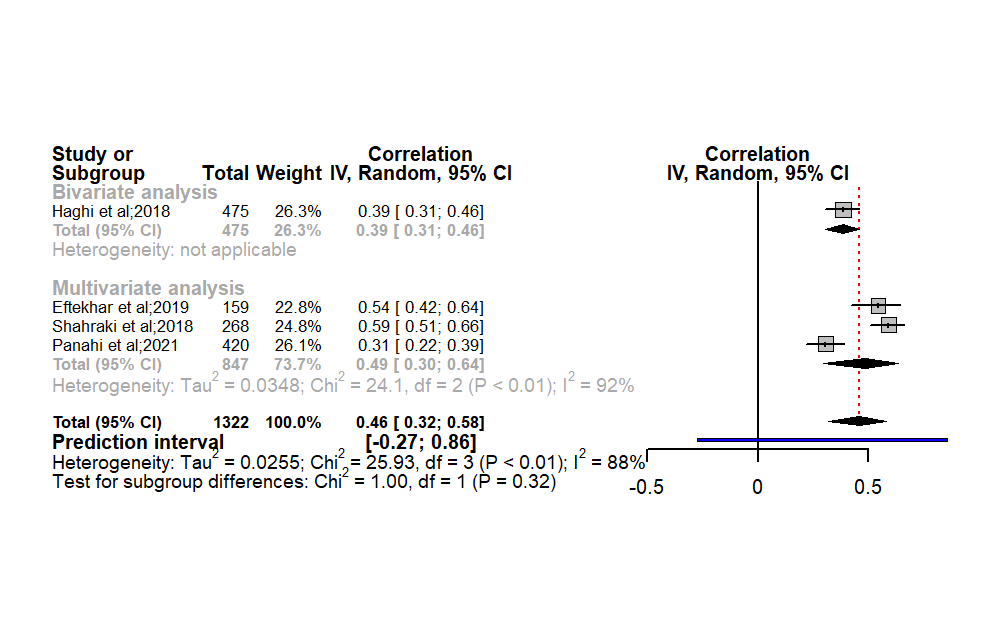


Figure S4: Subgroup analysis correlation coefficient with 95% confidence interval between sexual function and quality of sexual life by type of analysis


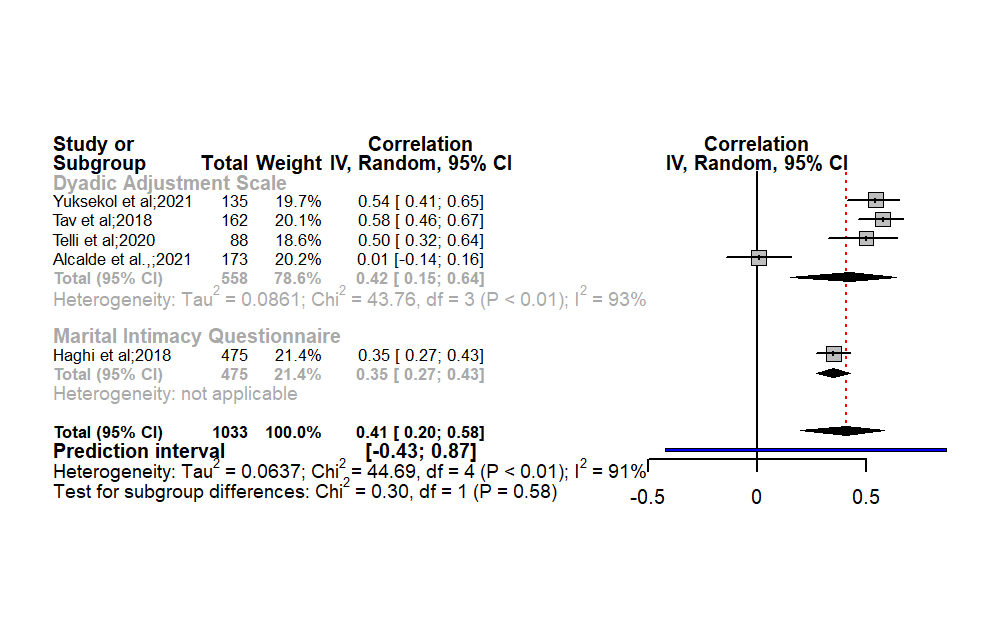


Figure S5: Subgroup analysis correlation coefficient with 95% confidence interval between quality of marital relation and quality of sexual life by type of scale


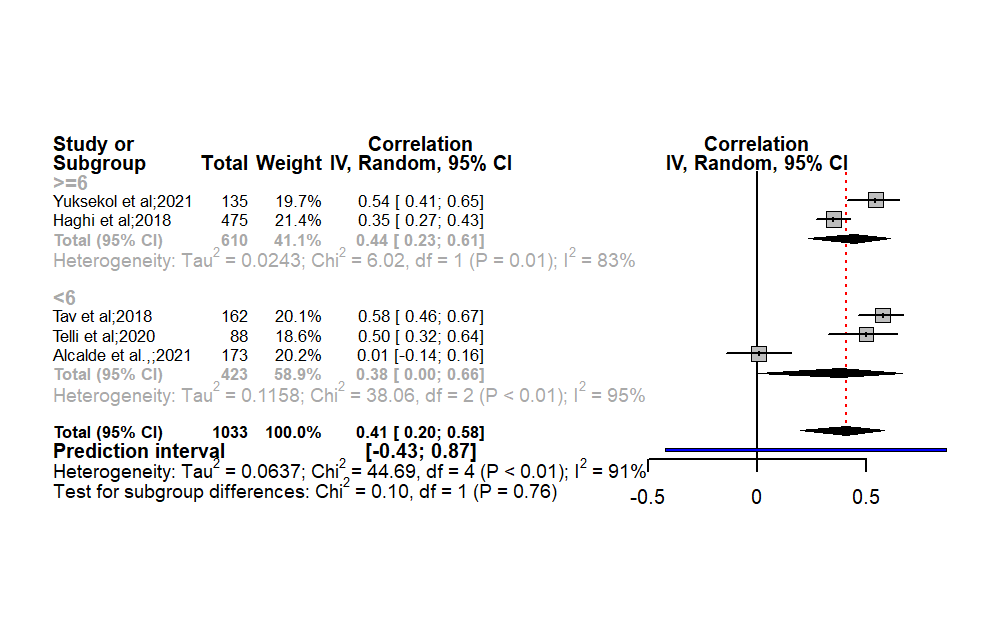


Figure S6: Subgroup analysis correlation coefficient with 95% confidence interval between quality of marital relation and quality of sexual life by quality assessment


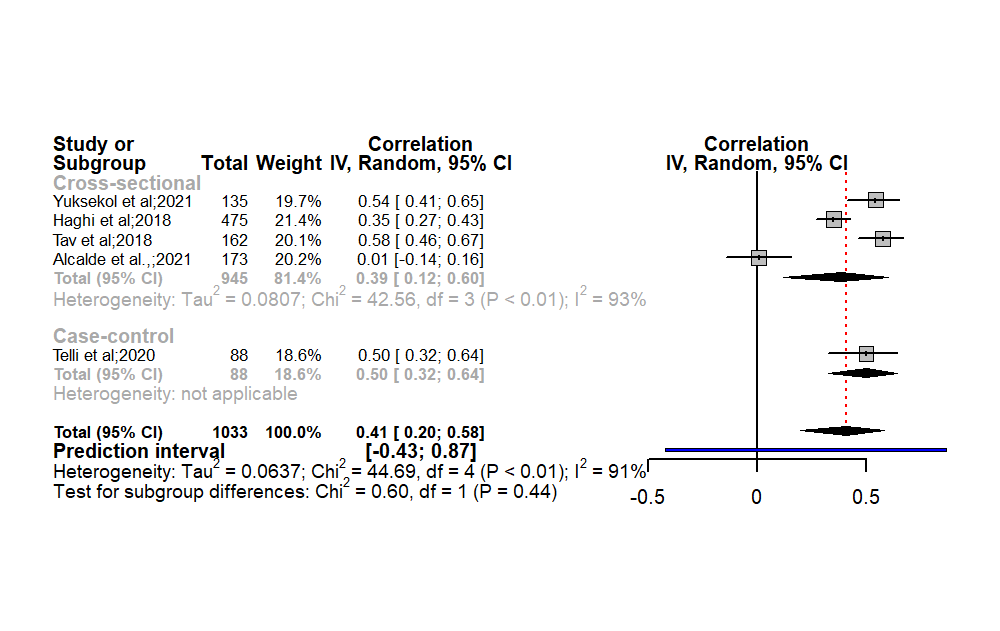


Figure S7: Subgroup analysis correlation coefficient with 95% confidence interval between quality of marital relation and quality of sexual life by study design


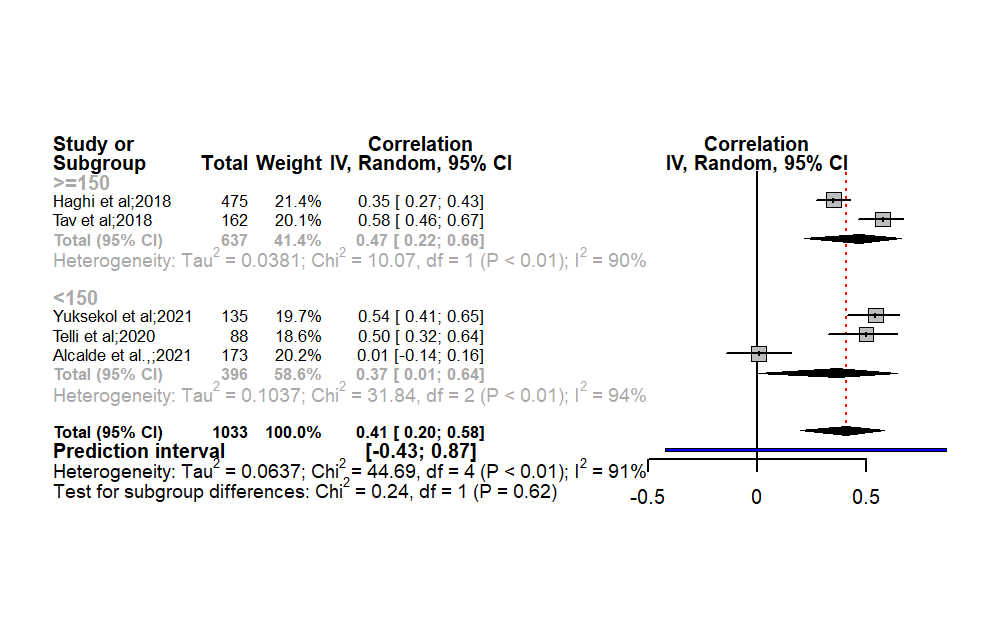


Figure S8: Subgroup analysis correlation coefficient with 95% confidence interval between quality of marital relation and quality of sexual life by sample size


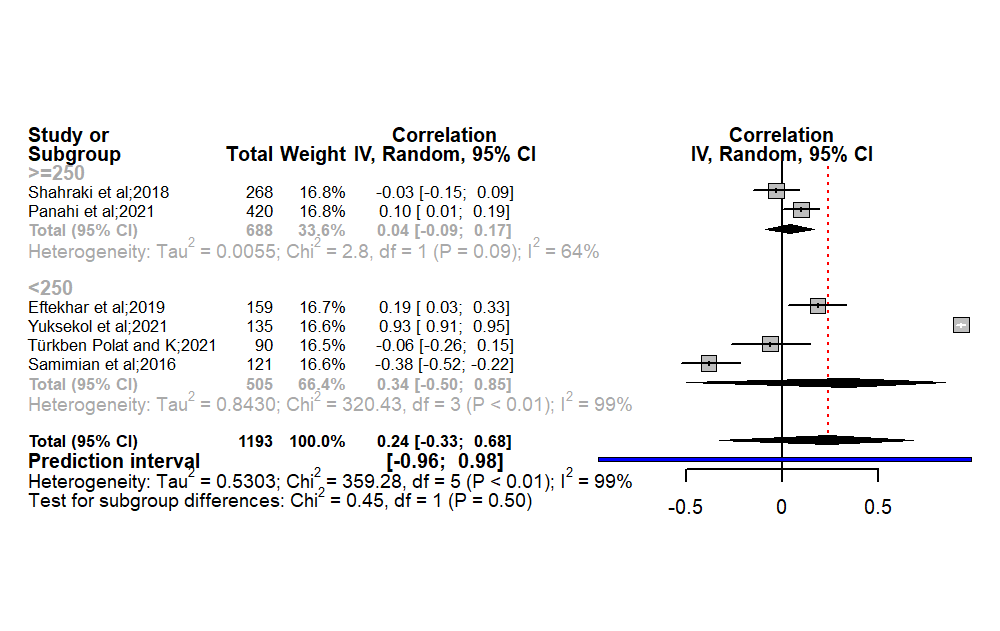


Figure S9: Subgroup analysis correlation coefficient with 95% confidence interval between duration of marriage and quality of sexual life by sample size


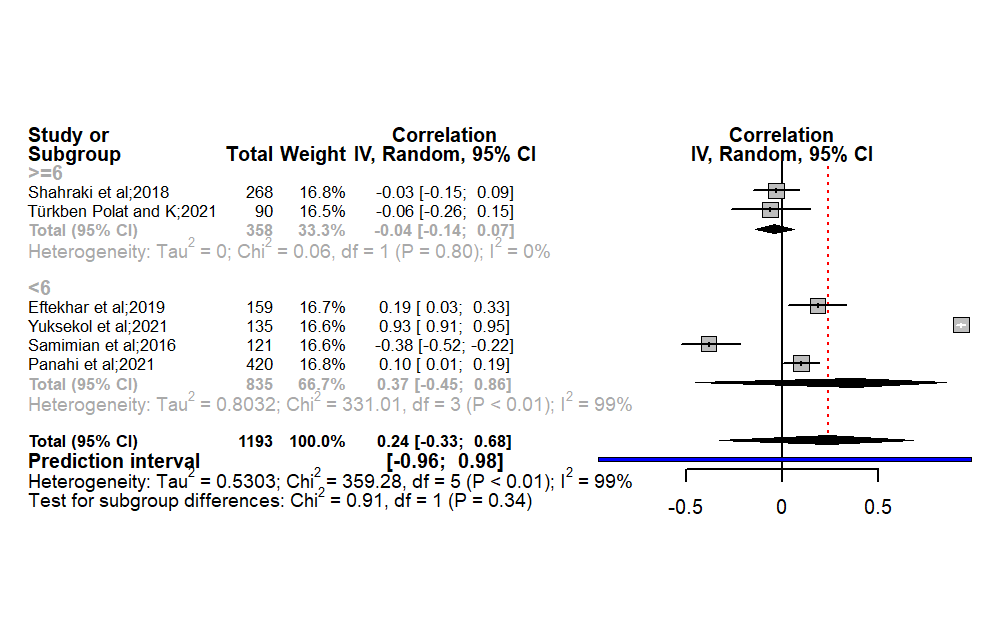


Figure S10: Subgroup analysis correlation coefficient with 95% confidence interval between duration of marriage and quality of sexual life by quality assessment


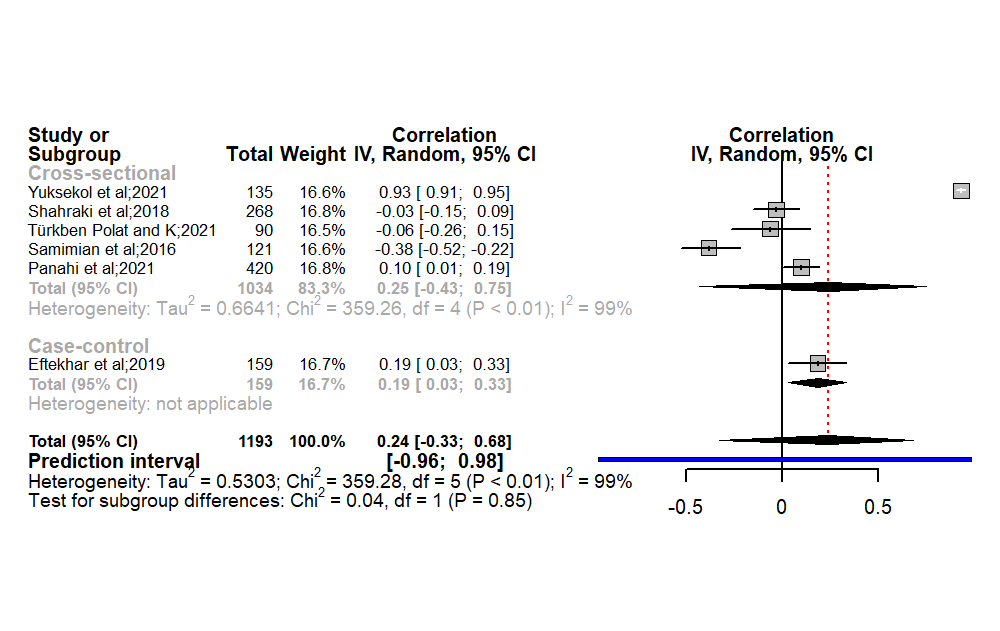


Figure S11: Subgroup analysis correlation coefficient with 95% confidence interval between duration of marriage and quality of sexual life by study design


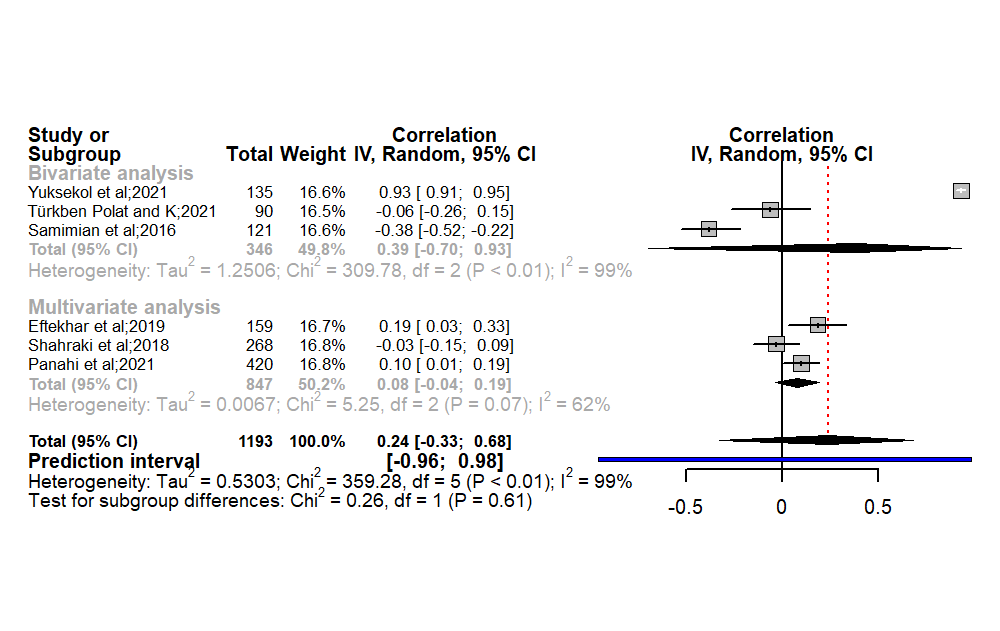


Figure S12: Subgroup analysis correlation coefficient with 95% confidence interval between duration of marriage and quality of sexual life by type of analysis
